# A revision of the fern genus *Oleandra* (Oleandraceae) in Asia

**DOI:** 10.3897/phytokeys.11.2955

**Published:** 2012-04-06

**Authors:** Peter H. Hovenkamp, Boon-Chuan Ho

**Affiliations:** 1 Netherlands Centre for Biodiversity Naturalis (section NHN), Leiden University, PO Box 9517, 2300 RA Leiden, The Netherlands; 2 Nees-Institut für Biodiversität der Pflanzen, Rheinische Friedrich-Wilhelms-Universität Bonn, Meckenheimer Allee 170, D-53115 Bonn, Germany

**Keywords:** *Oleandra*, systematics

## Abstract

The Asiatic species of *Oleandra* (Oleandraceae) are revised. We reduce a large number of species to *Oleandra neriiformis* and *Oleandra sibbaldii*, we provide a revised circumscription of *Oleandra cumingii* and *Oleandra undulata* and we establish the identity of *Oleandra vulpina*. In total, we recognize 9 species, with full synonymy, descriptions and distribution maps. A list of identifications is appended.

## Introduction

Virtually all authors who have dealt with the genus *Oleandra* Cav. have commented on its distinctness or naturalness. The shrubby growth form, particularly distinct in *Oleandra neriiformis* Cav., prompted Cavanilles (1799; 1802) not only to derive the genus name, but also the species name from *Nerium oleander* L. (Apocynaceae). From this it should be clear that he saw the aerial stems of *Oleandra neriiformis*, of which the forms with distinctly whorled fronds are indeed strongly reminiscent of branches of *Nerium oleander*. Subsequently, Swartz (1801; 1806) included *Oleandra* in *Aspidium* Sw., but Presl (1836) reinstated the genus, followed by Smith (1841; 1842), and the distinctness of the genus has not been questioned since, not even by Hooker (1862), and only one author has preferred to recognize *Oleandra* at subgeneric level, but not without some doubts (Splitgerber 1840, “An genus proprium? habitus distinctissimus!”).

Two genera have been described that are now universally included in *Oleandra*. Reinwardt (1825) described *Ophiopteris* Reinw., with the only species *Oleandra verticillata* Reinw., without distinguishing either from *Oleandra* or *Oleandra neriiformis*. Don (1825) distinguished *Neuronia* D. Don, with *Neuronia asplenioides* D.Don as only species.

The characters of *Oleandra* were summarized most recently by Smith et al. (2006) as “blades simple; leaves articulate, abscising cleanly upon senescence from pronounced phyllopodia; sori indusiate, indusia round-reniform; spores reniform, monolete”, while Tryon (2001) stressed the parallel veins, the unusual roots (“rhizophores”), the peltate scales and the articulate phyllopodia.

There is as much unanimity on the distinctness of the genus as there is uncertainty on the distinctness of species in *Oleandra*, and many authors who have dealt with the genus have commented on the difficulties of species delimitation. Smith et al. (2006) estimated a total number of c. 40 species, in which they follow many earlier authors, where the estimated number varies between 40 (“many species are similar in appearance”, Kramer 1990) to 50 (“closely related”, Pichi Sermolli 1965). However, Tryon (1997; 2001) reduced the number of American species to 4 (with one species indicated as of doubtful validity: “it may well be a variety …”) and of the Asiatic to 6. In addition, Pichi Sermolli (1965: “the delimitation of the species is not always easy, since some of them show a high degree of polymorphism”), distinguished no more than 5 species in Africa, and as his distinctions are sometimes subtle and based on limited material, the number should perhaps be reduced. A reasonable estimate of the number of species in *Oleandra* should accordingly be between 15 and 20. In this taxonomic treatment, we deal with the Asiatic species of *Oleandra*, extending the rather summary treatment in Tryon (2001), which was based on specimens in only two herbaria, and should be considered, according to the author, as “a prodromus that may be amplified”. After examining many more specimens in a much larger number of herbaria, we can only agree with Tryon and all others who have considered species delimitation in this group difficult.

## Morphology

Much of the variability between species in *Oleandra* is found in the rhizome, but as large parts of the rhizome are usually not preserved in herbarium collections, it is difficult to appreciate and describe this variability without field study. In the Asiatic species that we have seen, rhizomes may be relatively short-creeping, leading to more or less compact clumps of fronds, or more widely creeping. In the latter case, in some species the rhizome appears to be short-lived, decaying less than 1 m behind each growing point, which leads to stands with a scattered growth of plants. In others, the rhizome can be highly persistent, and stands may be extensive, with a dense growth of stems. In plants with this growth form, parts of the rhizome may also grow outwards or upwards from the substrate and form erect or drooping stems, here to be called “aerial” stems, that are often rootless. Branches are often opposite each other, and then both branches grow in the same direction, either downwards (positively geotropic) or upwards (negatively geotropic). Negatively geotropic branches can also be found on parts of the main rhizome where this starts to curve upwards, forming props that support the main stem. This is the shrubby growth form that is most often associated with the genus, although it occurs only in a minority of the species.

The anatomy shows a variably, but often very strongly developed peripheral sclerified sheath, a ground tissue with or without scattered sclerenchyma strands, and a dictyostele. When aerial parts develop, they tend to be more strongly sclerified.

The rhizome is covered with, usually persistent, rhizome scales with a peltate attachment. The scales are often strongly thickened near the attachment, and the margin can be nearly entire or densely set with woolly hairs (best visible in young scales) or sessile glands. The scales are strongly appressed or spreading to recurved - in the latter case the recurved parts tend to disappear on older rhizomes, giving the impression of a cover of short, appressed scales.

Roots arise mostly from the ventral side of the rhizome, and may be unbranched for a considerable length. The long unbranched parts have been described as rhizophores, but they were identified as real roots by Wetter (1951). Branching and the formation of root hairs appears to be limited to the parts of the root that are in contact with a suitable substrate, and thus the unbranched parts tend to be more evident in species that creep over the substrate, often at some distance, or over dense cushions of moss, than in species with a subterraneous rhizome.

Fronds arise on the rhizome without any apparent regularity, sometimes clustered, sometimes more regularly spread over the length of a creeping rhizome. They do not appear to grow in regular rows, but are inserted more or less dorsally on creeping stems, and often on all sides on aerial stems. At a variable position on the stipe, there is a distinct articulation point, where old fronds abscise cleanly, with a plane of dehiscence that is perpendicular to the stipe. The part below the abscission point might be called a phyllopodium, the part above it the stipe, but the upper part of the phyllopodium is in all structural details similar to the stipe, while basally it shows a gradual transition to the rhizome. A stipe-like phyllopodium like this is restricted, in ferns, to *Oleandra* and *Arthropteris* J. Sm., but in the latter genus, the articulation is distinctly much more oblique. In other ferns where fronds are articulated to the rhizome (Polypodiaceae, Davalliaceae), the part below the articulation is more clearly rhizomatous in structure and indument. In the most inclusive phylogenetic analyses available (Schuettpelz and Pryer 2007; Tsutsumi and Kato 2006), the optimization of the articulation of the fronds for the common ancestor of *Oleandra* and *Arthropteris* is ambiguous, so the homology of this type of articulation in *Oleandra* and *Arthropteris* is uncertain. Presence of an articulation in general (including the articulation of pinnae to the rachis) could well be an apomorphy for the crown clade of Eupolypods (Schuettpelz and Pryer 2007), related to the development of a climbing or epiphytic habit with a full reversal in the terrestrial Tectariaceae and a weaker reversal in the similarly terrestrial *Cyclopeltis* J.Sm.

The lamina is uniformly simple in all species, and varies little in shape, except in *Oleandra werneri* Rosenst., which is dimorphic, with the fertile fronds or parts of fronds strongly contracted. Sori are indusiate with a reniform indusium, and always separate. The indusia may be firm and persistent or shriveling and inconspicuous in older sori. The sori are borne dorsally and singly on the veins, at distances from the costa that vary strongly. Sporangia are long-stalked, often with a number of glandular hairs attached to the distal part of the stalk, just below the capsule. The capsule is of the common Polypodiales type.

### Spores

Figs 1–10

The morphology of the spores of *Oleandra* as shown with SEM has been illustrated by Liew (1977) and Tryon and Lugardon (1991), who illustrate the whole range of variability in perispore morphology in *Oleandra*.

**Figures 1–8.**
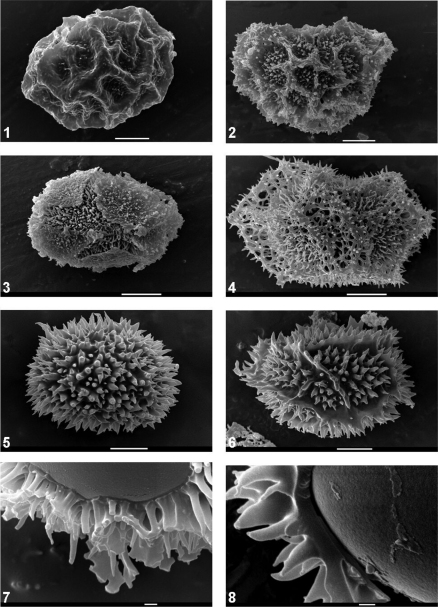
**1** *Oleandra cumingii*, perispore with broad folds, low warty ornamentation, not perforated. scale bar 10 µm. *Schmutz 6086* (L) **2** *Oleandra cumingii*, perispore with narrow wings, spiny ornamentation, few perforations. Scale bar 10 µm. *Ting & Shih 796* (L) **3** *Oleandra musifolia*, perispore with wings partly replaced by fissures, irregularly warty ornamentation, few perforations. Scale bar 10 µm. *Brooks s.n*. (L 0317430) **4** *Oleandra sibbaldii*, perispore highly perforated. Scale bar 10 µm. *Brass 29706* (L) **5** *Oleandra wallichii*, perispore spiny. Scale bar 10 µm. *De Haas 2622* (L) **6** *Oleandra wallichii*, perispore spiny, spines confluent into ridges. Scale bar 10 µm. *Van Steenis 20870* (L) **7** *Oleandra sibbaldii*. Perispore spines on a thin basal layer, exospore smooth, Scale bar 1 µm. *Elmer 11451* (L) **8** *Oleandra wallichii*, perispore with thick basal layer, exospore smooth. Scale bar 1 µm. *De Haas 2622* (L).

Two distinct types of perispore morphology can be distinguished. The first type occurs in all species studied except *Oleandra wallichii*. It is highly variable but the variation can be described in terms of just three parameters describing the folds, the ornamentation of the surface and the degree of perforation. This type shows a surface with a pattern of coarse folds, a variable ornamentation and a variable degree of perforation (“lophate”, Liew 1977). The folds may be broad (fig. 1), narrow (fig. 2), or replaced by fissures (fig. 3), the ornamentation ranges from warty (fig. 1) to densely spinose (fig. 2, 4). This surface may be perforated (fig. 4) or fissured to varying degrees, exposing an inner structure of numerous narrow cylindrical pillars c. 0.5 µm thick (fig. 7). These pillars are attached to a thin basal layer that adheres closely to the exospore. In the extreme case, the outer surface is so strongly perforated that the entire perispore consists of an open mesh (fig. 4). Within this range of variability, most species that could be studied with an adequate sample of specimens show variability in two or more of these parameters, and it is difficult either to subdivide this type or to identify character states that are characteristic for one species, although it appears that *Oleandra sibbaldii* has a more consistently highly perforated perispore than the other species.

The second type occurs exclusively in *Oleandra wallichii* (figs 5, 6, 8). Here the perispore is composed of a thicker basal layer and thicker, more conical spines (fig. 8) with a basal diameter of 1–2 µm, which are, mainly in the specimens from Taiwan, partly fused in irregular ridges (fig. 7). In this type there never is an outer layer overlaying this pattern of conical spines, and thus the entire perispore is massive.

**Figure 9.**
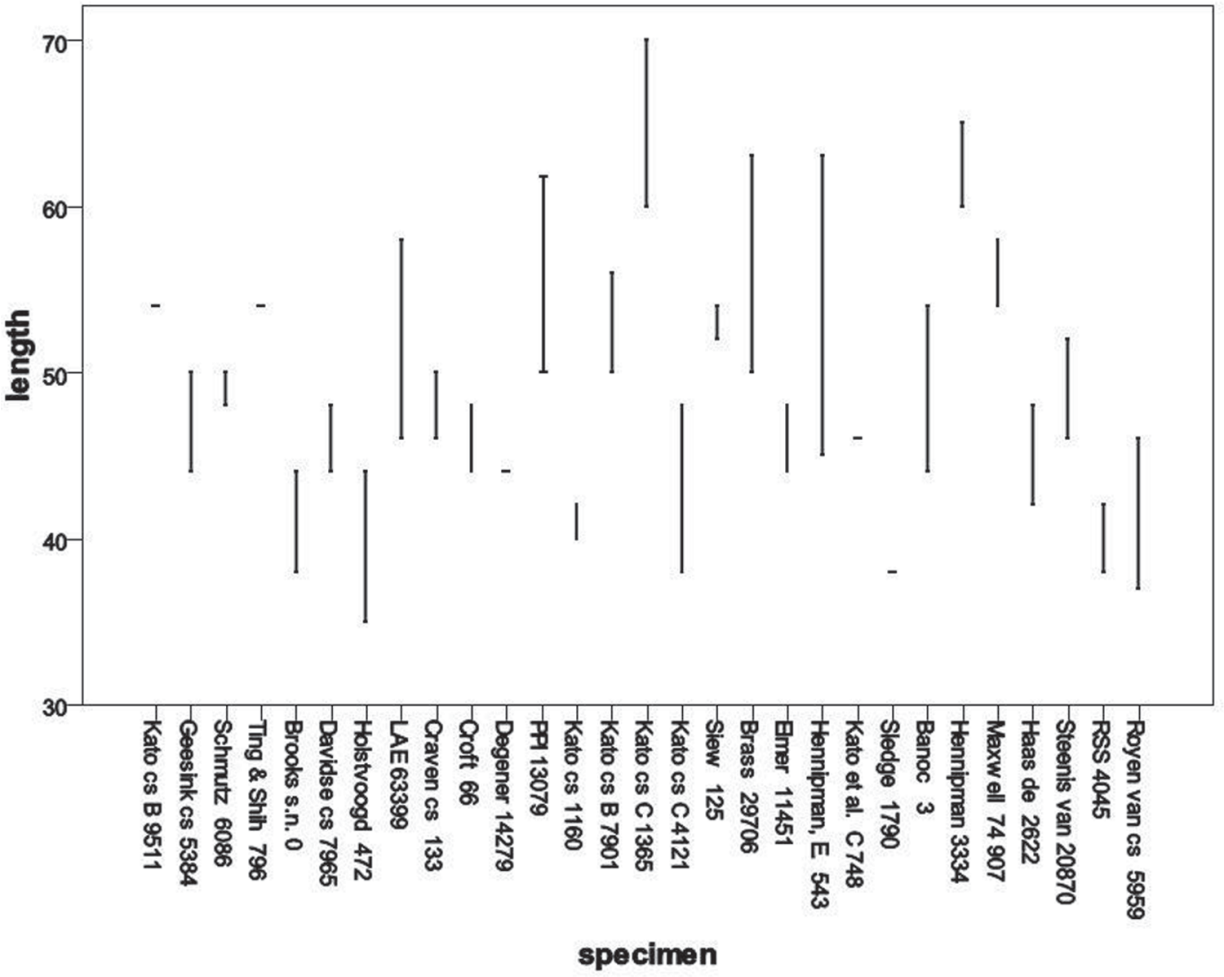
Distribution of spore lengths in *Oleandra*, arranged by specimen.

The exospore is smooth in all cases where it has been observed. Spore size variability was assessed based on SEM observations, with length of the spore measured including the perispore (Table 1, fig. 9). The limited data available do not allow a full statistical analysis, but it is clear that several species and even several specimens show a large variability in spore size. A similar variability in *Oleandra* from Southern Africa was found by Harmata and Kornás (1978).

**Table 1.** List of specimens (all L.) of which spores were studied (see figs. 9, 10).<br/>

**Specimen**	**Identification**	**number of spore measurements**
Kato et al. B 9511	*Oleandra coriacea*	2
Geesink & Santisuk 5384	*Oleandra cumingii*	3
Schmutz 6086	*Oleandra cumingii*	3
Ting & Shih 796	*Oleandra cumingii*	1
Davidse & Sumithraarachchi 7965	*Oleandra musifolia*	5
Holstvoogd 472	*Oleandra musifolia*	6
Brooks	*Oleandra musifolia*	4
Kato, M. et al. C 4121	*Oleandra neriiformis*	2
Kato, M. et al. C 1365	*Oleandra neriiformis*	4
Clunie et al. LAE 63399	*Oleandra neriiformis*	4
Degener 14279	*Oleandra neriiformis*	3
Siew 125	*Oleandra neriiformis*	3
Gaerlan et al. PPI 13079	*Oleandra neriiformis*	5
Kato et al. B 7901	*Oleandra neriiformis*	4
Kato et al. 1160	*Oleandra neriiformis*	5
Croft 66	*Oleandra neriiformis*	4
Craven & Schodde 133	*Oleandra neriiformis*	4
Brass 29706	*Oleandra sibbaldii*	6
Elmer 11451	*Oleandra sibbaldii*	2
Hennipman 5430	*Oleandra sibbaldii*	2
Kato et al. C 7480	*Oleandra sibbaldii*	1
Sledge 1790	*Oleandra sibbaldii*	1
Hennipman 3334	*Oleandra undulata*	4
Maxwell 74/907	*Oleandra undulata*	2
Banoc 3	*Oleandra undulata*	3
Van Steenis 20870	*Oleandra wallichii*	4
De Haas 2622	*Oleandra wallichii*	4
Van Royen & Sleumer 5959	*Oleandra werneri*	5
Braithwaite RSS 4045	*Oleandra werneri*	2

**Figure 10.**
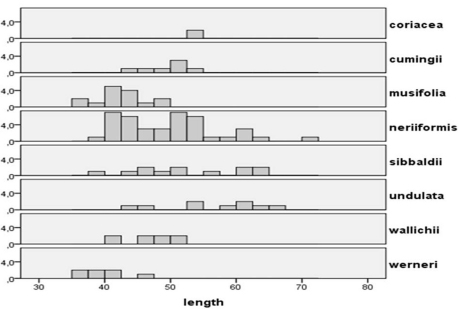
Distribution of spore lengths in *Oleandra*, arranged by species. Horizontal: spore length in µm, vertical: number of observed spores.

## Karyology

Data on chromosome numbers for species of *Oleandra* were summarized by Löve et al. (1977), who cite a basic number of x=41. Reports of n=40 (Kramer 1990) are based on a count that is acknowledged as uncertain by the authors (Manton and Sledge 1954) and that has not been confirmed by others.

Of the Asiatic species, *Oleandra neriiformis* (1 count, source Himalayas), *Oleandra musifolia* (Bl.) C. Presl (2 counts, Ceylon and Southern India) and *Oleandra wallichii* (Hook.) C. Presl (2 counts, Northern India) have been reported. The report of a tetraploid taxon in *Oleandra* is based on a count for unsourced material growing in Kew (presumably from Africa), and is complemented by reports of n=41 for another plant from West Tropical Africa. The large variation in spore size in *Oleandra* from Southern Africa was conjectured by Harmata and Kornás (1978) as due to the presence of different polyploidy levels, and this explanation would also fit well with the observed variability in morphology of *Oleandra* in Asia. However, actual confirmation of polyploidy in *Oleandra neriiformis* or any other Asiatic species is needed.

## Systematics

The simple morphology of *Oleandra* has made comparison with other ferns difficult, and its position has been judged to be with Dryopteridaceae or Davalliaceae, where it has been associated in particular with *Nephrolepis* Schott and *Arthropteris* (Copeland 1947, Holttum 1959, Nayar and Bajpai 1978). More recent views exclude *Nephrolepis* from this alliance (Kramer 1990, Tryon and Lugardon 1991, Tryon and Tryon 1982), and phylogenetic molecular studies (Kuo et al. 2011, Schuettpelz and Pryer 2007) have shown that these three genera do not form a clade, but that they are associated with different clades in the crown leptosporangiates.

## Taxonomic revision

This study is based on material from B, BISH, BM, BO, K, KEP, KLU, L, P, PE, PNH, MICH, SING, UC (abbreviations follow Index Herbariorum, Thiers 2011). All specimens cited were seen except where noted otherwise. Specimens seen only as on-line images (provided either directly through the database of the holding institute or via JSTOR, http://plants.jstor.org) are marked with *. The identification list (see Appendix 1) has been prepared with the help of BRAHMS (http://dps.plants.ox.ac.uk/bol/BRAHMS), and the distribution data with BRAHMS and DIVA-GIS (http://www.diva-gis.org/).

### Oleandra

http://species-id.net/wiki/Oleandra

Oleandra Cav., Anales Hist. Nat. 2: 115. 1799; Descr. Pl. (Cavanilles): 252. 1802. Lumbreras, Flora Montiber. 28: 19. 2004. *Aspidium* subg. *Oleandra* Splitg., Tijdschr. Natuurl. Gesch. Physiol. 7: 411. 1840. Type: *Oleandra neriiformis* Cav. (as “neriformis”).
Ophiopteris Reinw., Syll. Pl. Nov. 2: 3. 1825. Type: *Ophiopteris verticillata* Reinw., = *Oleandra neriiformis*.
Neuronia D.Don, Prodr. Fl. Nepal.: 6. 1825. Type: *Neuronia asplenioides* D.Don, =*Oleandra wallichii*.

#### Description.

Terrestrial, epilithic or epiphytic, creeping or scrambling ferns. Rhizome scaly, roots scattered, often with long rhizophore-like proximal parts, fronds scattered or in whorls, on stipe-like phyllopodia, dehiscing at a slightly thickened articulation point. Fronds stipitate, lamina simple, margin entire, veins distinct, somewhat raised on both sides, 1–2 ´ forked at or near the costa, costa often with narrow scales, lamina and veins often with acicular or capitate hairs. Sori in one, often irregular row on each side of the costa, with a more or less reniform, glabrous or hairy indusium. Sporangia stalked, stalk often with a number of sessile or stalked glands below the sporangium, sporangium body glabrous, spores monolete, perispore with broad wings, sometimes highly perforate, or echinate, massive.

#### Key to the 9 Asian species

**Table:** 

1	Rhizome with stiff, erect to pendent rootless aerial branches	2
–	Rhizome creeping	4
2	Fronds strongly dimorphic, scattered on the rhizome or somewhat clustered on short side branches	9 *Oleandra werneri*
–	Fronds monomorphic or slightly dimorphic, often in whorls of 5–10 fronds	3
3	Lamina thick, coriaceous, costa with copious, conspicuous, 3–4 mm long pale to brown scales on the abaxial surface	1 *Oleandra coriacea*
–	Lamina thin, papyraceous when dry, costa mostly with few or inconspicuous scales on the abaxial surface	4 *Oleandra neriiformis*
4	Rhizome in older parts not entirely covered with scales; scales with squarrose acumen and entire or distinctly glandular margin	5
–	Rhizome entirely covered with overlapping scales; scales with appressed or spreading apex, usually with non-glandular cilia	6
5	Rhizome scales with gradually narrowed apex, stipes 0.5–4.5 cm long, costa and stipe often with distinct dark colouration on the abaxial surface	5 *Oleandra sibbaldii*
–	Rhizome scales with abruptly narrowed apex, stipe to 2–3 mm long, costa and stipe without dark colouration on the abaxial surface	7 *Oleandra vulpina*
6	Phyllopodia , inconspicuous, 2–5 mm, rarely 1 cm long, much shorter than the stipe, sori mostly in a closely costal single row	7
–	Phyllopodia, conspicuous, 2–10 cm long, often as long as or longer than the stipe, position of sori costal to medial	8
7	Rhizome scales squarrose, costa and stipe usually with dark colouration, frond apex distinctly apiculate	8 *Oleandra wallichii*
–	Rhizome scales appressed, costa and stipe without dark colouration, frond apex acute to acuminate	3 *Oleandra musifolia*
8	Rhizome scales spreading, long triangular with a wide acumen, brown, central part not conspicuously thickened, roots branching with root hairs over their entire length; lamina glabrous on upper surface and margin	6 *Oleandra undulata*
–	Rhizome scales appressed to spreading, narrowly ovate-lanceolate with long narrow acumen, central part dark, thickened, roots often with glabrous, unbranched part (“rhizophore”), lamina hairy or glabrous	2 *Oleandra cumingii*

### 1. Oleandra coriacea

http://species-id.net/wiki/Oleandra_coriacea

Map 1 fig. 11 d–f

Oleandra coriacea Copel. J. Straits Branch Roy. Asiat. Soc. 63: 72. 1912. Type: MALAYSIA. Borneo:Moulton s.n.(SAR? not seen).

#### Description. 

*Rhizome* with creeping parts unknown, aerial stems unbranched and rootless, 2–3 mm thick (when dry), not white waxy, in cross-section with a distinct sclerified peripheral sheath and few, scattered sclerified strands, phyllopodia in weak to distinct clusters of 4–6, short, usually less than 5 mm long. *Scales* persistently covering the rhizome, peltate, 5–70 × 0.5–1 mm, appressed (sometimes spreading), dark, shining with pale to brown margin and acumen, margin ciliate especially when young. *Fronds* monomorphic; stipe 1–1.5 cm long, without dark colouration, with up to 1 mm long glandular hairs and often small, appressed scales; lamina 13–30 × 1.2–3.3 cm, linear, base narrowly cuneate to rounded, apex acuminate to caudate with cauda to 1.5 cm, texture coriaceous; costa and veins on lower surface densely hairy with up to 1 mm long, acicular hairs, costa without dark colouration, with copious, conspicuous, 3–4 mm long pale to brown scales, upper surface more glabrous, mostly hairy on the costa only, with similar hairs and with less copious scales. Sori in a single more or less irregular medial row, separated from the costa by a 2–7 mm wide sterile zone, indusium distinct, c. 1.5 mm wide, most often glandular. Sporangial stalk with glands below the sporangium. Spores with coarse confluent ridges, areolae with short pointed excrescences, perispore hollow, with internal baculae, outer layer distinctly perforated.

**Map 1.**
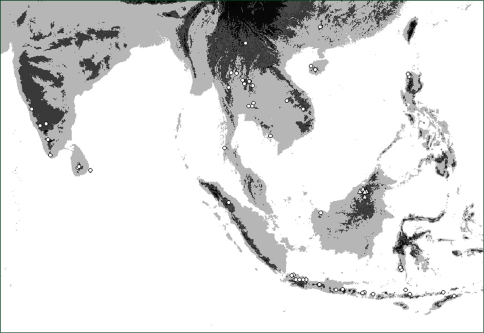
Distribution of *Oleandra coriacea* (triangles), *Oleandra musifolia* (circles).

**Figure 11.**
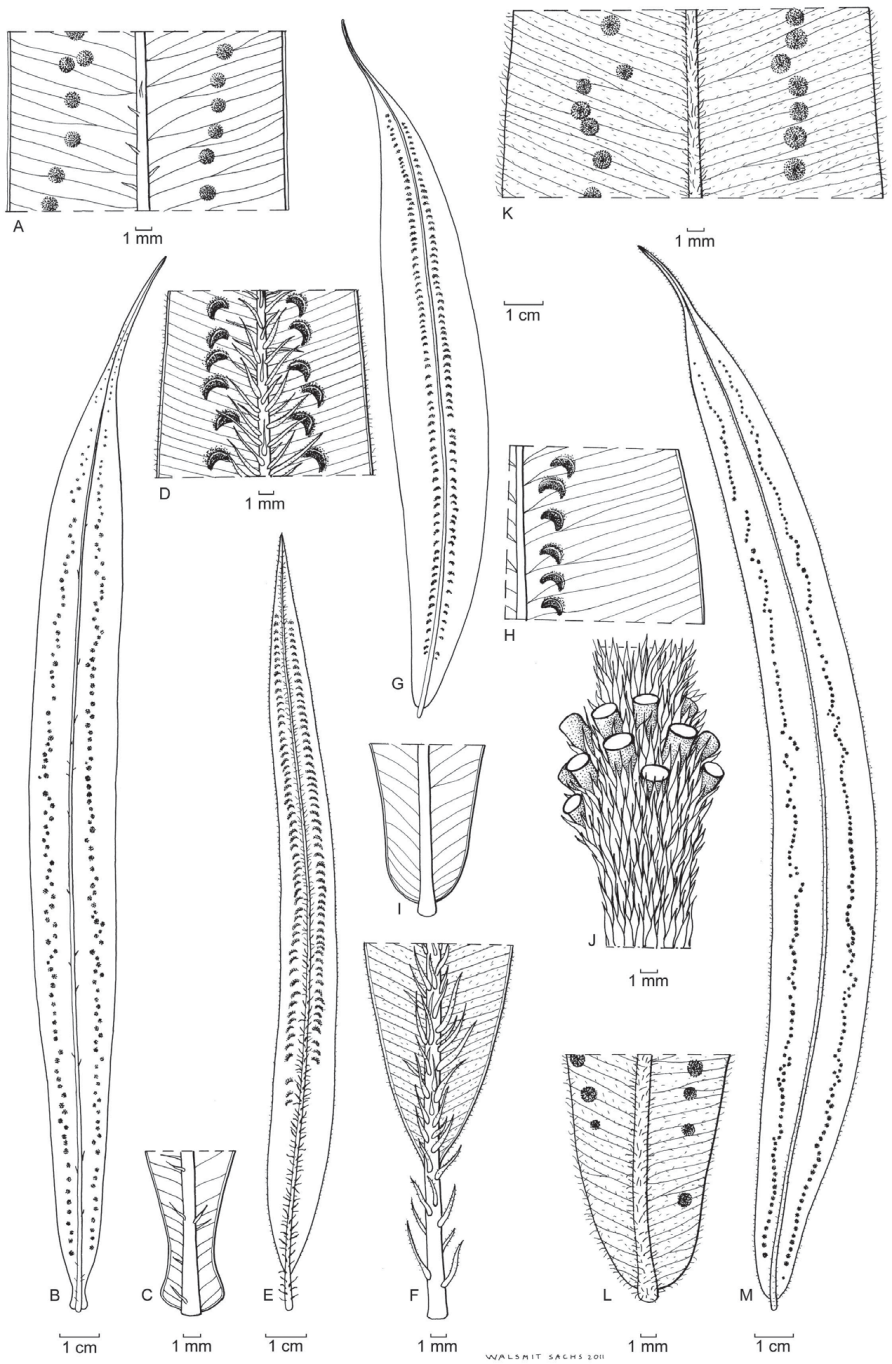
*Oleandra neriiformis* (**a–c, g–m**), *Oleandra coriacea* (**d–f**). **a** middle part of lamina, medial sori with indistinct indusia **b** entire lamina **c** lyrate lamina base with stipe articulation immediately below lamina base: *Brass 2301*6 (New Guinea) **d** middle part of lamina, with conspicuous scales on midrib and sori with persistent indusia **e** entire lamina **f** lamina base with elongated stipe: *Yahud et al SAR 88396* (Borneo) **g** entire lamina with costal sori **h** middle part of lamina, sori costal with persistent indusia **i** rounded lamina base with short stipe **j** whorl of short phyllopodia on aerial stem: *Chew, Corner & Stainton 298* (Borneo) **k** middle part of lamina sori with inconspicuous indusium **l** rounded lamina base with short stipe **m** lamina: *Copeland s.n. 29 jan 1933* (Philippines)., All views of lamina showing abaxial surface; all specimens L.

#### Distribution.

Brunei, Indonesia: Kalimantan Timur; Malaysia: Sarawak.

E

#### cology.

Terrestrial in montane forest, mainly on ridges and in summit vegetation, 1100–2200 m.

#### Discussion.

Creeping and rooting parts of the rhizome are absent in all collections seen, but presumably present, as in *Oleandra neriiformis*. The lamina of *Oleandra coriacea* is indeed much more coriaceous than *Oleandra neriiformis*, and has copious large pale costal scales and long hairs especially on lower surface. The sori are consistently medial, with firm indusia that are often clearly glandular on their surface.

### 2. Oleandra cumingii

http://species-id.net/wiki/Oleandra_cumingii

Map 2

Oleandra cumingii J.Sm., J. Bot. (Hooker) 4: 413. 1842. C.Presl, Epimel. Bot.: 41. 1851. Fée, Mém. Foug., 5. Gen. Filic.: 304. 1852. Hook., Sp. Fil.: 158. 1862. Baker, Syn. Fil. (Hooker & Baker): 303. 1868. Copel., Polypod. Phil. Isl.: 49. 1905. Copel., Fern Flora of the Philippines: 184. 1958. Ching, Fl. Reipubl. Popularis Sin. 2: 324. 1959. X.C.Zhang, Ching Mem. Vol.: 91. 1999. Type: PHILIPPINES. Luzon: Cuming 60. (holotype: K; isotypes: B, BM, SING, US*),
Oleandra macrocarpa C.Presl, Epimel. Bot.: 41. 1851. Fée, Mém. Foug., 5. Gen. Filic.: 304. 1852. Holttum, Novit. Bot. Inst. Horto Bot. Univ. Carol.: 43. Type: PHILIPPINES. Luzon: Cuming 60 p.p. (Holotype: PRC, teste Holttum; isotypes: B, BM, K, SING, US*).
Oleandra chinensis Hance, Ann. Sci. Nat. (Paris) 18: 238. 1862. Type: CHINA. Guangdong: Si Chu Shan, Parry s.n., herb. Hance 9408 (holotype: K; isotype BM).
Oleandra scandens Copel., Philipp. J. Sci. 46: 218. 1931., Fern Flora of the Philippines: 184. 1958. Type: PHILIPPINES. Baguio: Elmer 6513 (holotype: US*).
Oleandra intermedia Ching, Bull. Fan Mem. Inst. Biol. 11: 187. 1931. Ching, Fl. Reipubl. Popularis Sin. 2: 323. 1959. X.C.Zhang, Ching Mem. Vol.: 92. 1999. Type: CHINA. Yunnan: Henry 9484c (holotype: K; isotypes: B, BM, MO*, PNH, US*).
Oleandra cumingii var. *longipes* Hook., Sp. Fil.: 158. 1860. *Oleandra longipes* Ching, Lingnan Sci. J. 12: 158. 1933. Type: BIRMA. Mergui: Parish 59 (holotype: K).
Oleandra cantonensis Ching, Fl. Reipubl. Popularis Sin. 2: 324, 378. 1959. Type: CHINA. Guangdong: Canton, Peiyinshan,.Y. Liang 60252 (holotype: PE).
Oleandra yunnanensis Ching, Fl. Reipubl. Popularis Sin. 2: 325. 1959. Type: CHINA. Yunnan: anon. s.n. (holotype: PE).
Oleandra guangxiensis S.L. Mo & Y.C. Zhong, Guihaia 7: 289. 1987. Type: CHINA. Giangxi: Damingshan, Guangxi Forest ecology group 84422 ( holotype: IBY, not seen; isotype: PE).

#### Description.

Rhizome short- to long creeping, 3–8 mm thick, sometimes white waxy in the older parts, little branched and not forming extensive stands, in cross-section with or without scattered sclerified strands; roots scattered, sometimes with unbranched aerial parts; phyllopodia scattered or more or less tufted, (1–)3–10 cm long. Scales persistently covering the rhizome, peltate, 4–9 × 0.5–1.5 mm, appressed, acumen with dark center and lighter acumen and margin, margin ciliate especially when young. Fronds monomorphic, stipe 2–12 cm long, without dark colouration, glabrous or hairy with catenate to acicular up to 2 mm long hairs, lamina 12- 40 × 2–4.5 cm, base narrowly cuneate to truncate, apex acute to long-acuminate, texture thin-chartaceous, both surfaces and margin with catenate or acicular hairs 0.2–1 mm long, usually more densely on lower surface, costa without dark colouration, on lower surface without or with few, pale to dark scales. Sori close to or scattered up to 2 mm from the costa, indusium distinct, 1–2 mm wide, densely hairy with short or long hairs. Sporangial stalk with glands below the sporangium. Spores with broad or narrow confluent ridges, surface variably ornamented with small pustules to narrow spines, perispore hollow, with internal baculae, outer layer not or finely perforated, sometimes fissured along the ridges.

#### Distribution.

China: Yunnan, Guangdong, Guangxi, Guizhou; Laos; Thailand (Peninsular); Malaysia: Peninsular Malaysia; Indonesia: Flores; Timor Leste; Philippines: Luzon.

**Map 2.**
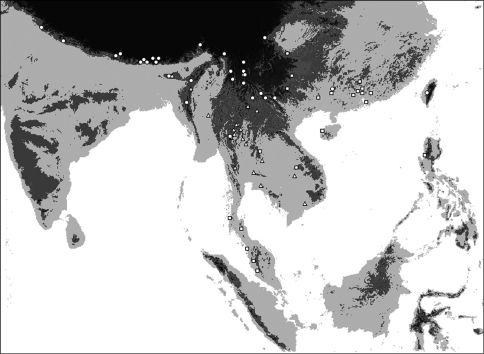
Distribution of *Oleandra cumingii* (squares), *Oleandra undulata* (triangles), *Oleandra wallichii* (circles).

#### Ecology.

Terrestrial or on rocks, cliffs and roadsides in open forest, sea level to c. 1200 m.

#### Discussion.

Contrary to Holttum (1968a), *Oleandra cumingii* J. Sm. is not a *nomen nudum*, but provided with an adequate diagnosis. *Cuming 60* is distributed in several herbaria with many duplicates, and it is impossible to tell which ones should be considered isotypes of *Oleandra cumingii* and which ones of *Oleandra macrocarpa*.

*Oleandra cumingii* is a variable assemblage of fairly widely scattered forms, in many characters intermediate between *Oleandra undulata* and *Oleandra musifolia*. From *Oleandra undulata* it can be distinguished by the more narrowly elongated, appressed rhizome scales with a more or less thickened costa in the acumen, and by the often hairy upper surface of the lamina. From *Oleandra musifolia*, *Oleandra cumingii* differs in the elongated phyllopodia and the distinctly tomentose indusia and lower surface of the lamina. The differences with either species, however, are often slight and bridged by specimens with an intermediate character combination. This may indicate that *Oleandra cumingii* is a hybrid or a hybrid swarm between the two other species, but failing positive evidence for that interpretation, we prefer to regard it here as a separate species.

### 3. Oleandra musifolia

http://species-id.net/wiki/Oleandra_musifolia

Map 1

Oleandra musifolia Blume, Enum. Pl. Javae: 141. 1828. (as *Oleandra musaefolium*). *Oleandra musaefolia* C.Presl, Epimel. Bot.: 42. 1851. Fée, Mém. Foug., 5. Gen. Filic.: 304. 1852. Ching, Fl. Reipubl. Popularis Sin. 2: 321. 1959. *Oleandra musifolia* C.Chr., Index Filic.: 466. 1906. Backer & Posth., Varenfl. Jav.: 87. 1939. X.C.Zhang, Ching Mem. Vol.: 94. 1999. R.M.Tryon, Rhodora 102: 434, fig. 4. 2001. Type: INDONESIA. Java: Gedeh, Blume s.n. (holotype: L).
Aspidium moritzii Kunze, Bot. Zeitung (Berlin) 6: 238. 1848., Kunze, Bot. Zeitung (Berlin) 9: 348. 1851. *Oleandra moritzii* C.Presl, Epimel. Bot.: 42. 1851. Fée, Mém. Foug., 5. Gen. Filic.: 304. 1852. Type: INDONESIA. Java: Zollinger 1306B (L, lectotype L 0317415, here selected).
Oleandra geniculata Alderw., Bull. Jard. Bot. Buitenzorg Ser. 2: 23. 1914. Type: INDONESIA. Java: Docters van Leeuwen s.n.(holotype BO? not seen).
Oleandra benguetensis Copel., Philipp. J. Sci. 46: 217. 1931. Fern Flora of the Philippines: 183. 1958. Type: PHILIPPINES. Baguio: Elmer 6286(holotype: US*).
Oleandra whangii Ching, Bull. Dept. Biol. Sun Yatsen Univ. 6: 23. 1933. Type: CHINA. Guangxi: Sin & Whang 300 (holotype: SYS, not seen; isotypes: NY, PE, BM).
Oleandra hainanensis Ching, Acta Phytotax. Sin. 8: 141, pl. 20 fig. 17. 1959. 141; Ching, Fl. Reipubl. Popularis Sin. 2: 322. 1959. X.C.Zhang, Ching Mem. Vol.: 91. 1999. Type: CHINA. Hainan: S.K. Lau 27326 (holotype: PE).
Aspidium lomatopus Kunze, Bot. Zeitung (Berlin) 6: 238. 1848. *Oleandra lomatopus* C.Presl, Epimel. Bot.: 43. 1851. Fée, Mém. Foug., 5. Gen. Filic.: 304. 1852. Type: INDONESIA. Java: Zollinger s.n. (lectotype: K, here selected).
Oleandra neriiformis auct. non Cav.:Bell, Flora of Australia 48: 446. 1998.

#### Description.

*Rhizome* creeping, 5–8 mm thick, white waxy in the older parts, often supported above the substrate by unbranched stilt-like roots, dorsiventrally flattened, (strongly compressed, 4–6 mm wide when dry), with up to 10 cm long, curved internodes (only occasionally straight and then often much longer) terminating in a cluster of a few short phyllopodia, usually less than 5 (–15) mm long, often hidden by the scales, rhizome often innovating just below this cluster, lateral branches usually basal on the internodes, in opposite pairs; all parts in cross-section without or with few sclerified strands, roots scattered, with long unbranched aerial parts. *Scales* persistently covering the rhizome, peltate, 5–10 × 1–1.5 mm, appressed, with dark center and lighter brown acumen and margin, margin ciliate especially when young and with sessile glands. *Fronds* monomorphic, stipe 0.5–4 cm long, without dark colouration, with short, glandular hairs; lamina to 60 × 4.2 cm, linear, base cuneate to truncate or more or less rounded, apex acute to long-acuminate, texture thin-chartaceous, both surfaces with catenate, often glandular hairs 0.2–0.5 mm long; costa without dark colouration, on lower surface with inconspicuous, 1–3 mm long brown scales. *Sori* mostly in a single regular row close to the costa, sometimes more scattered over a 2–5 mm wide zone close to the costa or at a distance of up to 3 mm, indusium distinct, 1.5–2 mm wide, glabrous or glandular, sometimes setose. Sporangial stalk with glands below the sporangium. Spores with coarse confluent ridges, areolae with short pointed excrescences, perispore hollow, with internal baculae, outer layer not or hardly perforated.

#### Distribution.

South China, Southern India, Sri Lanka, Thailand, Indonesia: Java, Lesser Sunda Islands, Sulawesi; Philippines: Luzon, Australia: Queensland.

#### Ecology.

Mostly terrestrial or on rocks, less often as low trunk epiphyte, in various types of forests, often disturbed, sea level to c. 2000 m.

#### Discussion.

ICN 60.8 specifies that the spelling of the original epithet “musaefolia” should be corrected to “musifolia”.

*Aspidium moritzii* is one of the two species distinguished by Kunze (1848) among the collections under Zollinger 1306 (see also discussion under *Oleandra neriiformis*). The description conforms to *Oleandra musifolia*, and the specimen selected as lectotype for *Aspidium moritzii* is conform to the original description, and bears a label “Aspidium moritzi 1306B”. *Aspidium lomatopus* is based on a description and name in Kunze (1848) that was evidently considered preliminary by Kunze, as he did not refer to this species in his later account (Kunze 1851). A specimen in K is labeled “Zollinger 1306 b Aspidium moritzii Kze”, with a separate label “Oleandra lomatopus” in what may be Kunze’s hand. It is *Oleandra musifolia*, and is here selected as lectotype.

*Oleandra musifolia* is variable in the density of hairs is variable, and the distance of the sori to the costa. It can be distinguished from *Oleandra cumingii* mainly by the short phyllopodia (longer in *Oleandra cumingii*). Incomplete collections are easily confused also with *Oleandra neriiformis* but can often be recognized by the flatter, softer rhizome without sclerenchyma strands (rhizome more rigid, rounded, with sclerenchyma strands in *Oleandra neriiformis*) and by the catenate hairs with capitate or glandular apex (more acicular, not capitate in *Oleandra neriiformis*). Distinguishing these two species on basis of juvenile material, however, is difficult, and often impossible.

### 4. Oleandra neriiformis

http://species-id.net/wiki/Oleandra_neriiformis

**Map 3.**
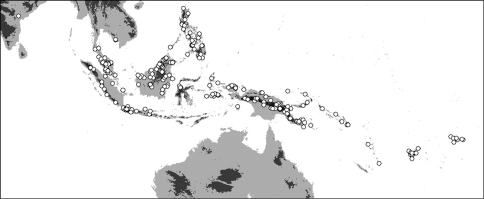
Distribution of *Oleandra neriiformis*.

Map 3 fig. 11 a–c, g–m 12

Oleandra neriiformis Cav., Anales Hist. Nat. 2: 115. 1799. (as “*neriformis*”); Descr. Pl. (Cavanilles): 252. 1802. C.Presl, Tent. Pterid.: 78. 1836. (as “*neriifolia”)*; C.Presl, Epimel. Bot.: 42. 1851. Fée, Mém. Foug., 5. Gen. Filic.: 304. 1852. Hook., Sp. Fil.: 156. 1862. Copel., Polypod. Phil. Isl.: 49. 1905. Backer & Posth., Varenfl. Jav.: 87. 1939. Copel., Philipp. J. Sci. 73: 346. 1940. C.Chr., Bull. Bernice P. Bishop Mus. 177 45. 1943. Copel., Fern Flora of the Philippines: 182. 1958. Brownlie, The Pteridophyte Flora of Fiji: 156. 1977. M.Kato, J. Fac. Sci. Univ. Tokyo, Sect. 3, Bot. 14: 240. 1989. R.M.Tryon, Rhodora 102: 430. 2001. *Aspidium neriiforme* Sw., Syn. Fil. (Swartz) 42. 1806. Willd., Sp. Pl., ed. 4 [Willdenow] 5: 212. 1810. Blume, Enum. Pl. Javae: 140. 1828. Gariletti, Fontqueria 38: 40. 1993. Type: PHILIPPINES. Mauban: Née s.n. (holotype: MA 476029, teste Gariletti, not seen).
Aspidium pistillare Sw., J. Bot. (Schrader) 1800: 30. 1801. *Oleandra pistillaris* C.Chr., Index Filic., Suppl. Tertium pro Annis 1917-1933: 132. 1934. Holttum, Rev. Fl. Mal. 2: 386. 1954. X.C.Zhang, Ching Mem. Vol.: 91. 1999. Type: INDONESIA. Java: Unknown s.n. (holotype S, not seen) (teste Sw. 1806).
Ophiopteris verticillata Reinw., Syll. Pl. Nov. 2: 3. 1825. Type: INDONESIA. Java: Reinwardt (?) s.n.(L).
Aspidium bantamense Blume, Enum. Pl. Javae: 141. 1828. *Oleandra bantamense* Kunze, Bot. Zeitung (Berlin) 9: 349. 1851. Type: INDONESIA. Java: Bantam, anon. (Kuhl & van Hasselt?) s.n.(Holotype: L).
Aspidium micranthum Blume, Enum. Pl. Javae: 141. 1828. *Oleandra micrantha* Kunze, Bot. Zeitung (Berlin) 9: 349. 1851. Type: INDONESIA. Java, Salak.: anon. (Kuhl & van Hasselt?) s.n.(holotype: L).
Aspidium salaccense Blume, Enum. Pl. Javae: 140. 1828. *Aspidium neriiforme* var. *salaccense* Blume, Enum. Pl. Javae: Add. et emend. 1828. *Oleandra neriiformis* var. *salaccensis* Kunze, Bot. Zeitung (Berlin) 9: 348. 1851. Type: INDONESIA. Java: Blume s.n. (holotype: L).
Blechnum colubrinum Blanco, Fl. Filip. [F.M. Blanco]: 834. 1837. *Oleandra colubrina* Copel., Polypod. Phil. Isl.: 48. 1905. Copel., Fragm. Fl. Philipp. 3 179. 1905. Merrill, Sp. Blancoan.: 43. 1918. Copel., Fern Flora of the Philippines: 181. 1958. M.Kato, J. Fac. Sci. Univ. Tokyo, Sect. 3, Bot. 14: 240. 1989. Type: PHILIPPINES. Unknown.
Aspidium phyllarthron Kunze, Bot. Zeitung (Berlin) 6: 237. 1848. *Oleandra phyllarthron* C.Presl, Epimel. Bot.: 42. 1851. Kunze, Bot. Zeitung (Berlin) 9: 349. 1851. Type: INDONESIA. Java: Zollinger 1306 (lectotype: L 0317564, here selected) (see discussion).
Oleandra hirtella Kunze, Farnkräuter: 70, pl. 129. 1847. Fée, Mém. Foug., 5. Gen. Filic.: 304. 1852. *Oleandra neriiformis* var. *hirtella* Hook., Sp. Fil.: 156. 1862.Type : INDONESIA. Java : Miquel? s.n. (holotype L?, not found).
Oleandra mollis C.Presl, Epimel. Bot.: 41. 1851. Fée, Mém. Foug., 5. Gen. Filic.: 304. 1852. Type: PHILIPPINES. Luzon: Cuming 94 p.p. (holotype: PRC, not seen; isotypes: BM, L, SING, US*).
Oleandra neriiformis var. *brachypus* Hook., Sp. Fil.: 156. 1862. Type: UNKNOWN. Malay Archipelago: Norris s.n. (holotype: K, not seen).
Oleandra cumingii var. *tahitense* Hook., Sp. Fil.: 159. 1862. Type: FRENCH POLYNESIA. Tahiti: Greville s.n. (holotype: K, not seen).
Oleandra ciliata Kuhn, Linnaea 36: 126. 1869. Type: VANUATU. Aneiteum: Cuming 48 (holotype: B).
Oleandra cuspidata Baker in Becc., Malesia 3: 44. 1886. Copel., Philipp. J. Sci. 73: 346. 1940. M.Kato, J. Fac. Sci. Univ. Tokyo, Sect. 3, Bot. 14: 239. 1989. Type: INDONESIA. New Guinea: Arfak, Beccari s.n. (holotype: K).
Oleandra colubrina var. *nitida* Copel., Philipp. J. Sci., C 3: 33. 1908. *Oleandra nitida* Copel., Fern Flora of the Philippines: 181. 1958. Amoroso & Pava, Philipp. J. Sci. 120: 423, 437. 1991. Type: PHILIPPINES. Mindanao: Mt. Apo, Copeland 1474(lectotype: US*, here selected).
Oleandra colubrina var. *membranacea* Copel., Philipp. J. Sci., C 3: 32. 1908. Type: PHILIPPINES. Luzon: Mt. Maquiling, Copeland PPE57 (holotype MICH; isotypes K, PNH, UC).
Oleandra oblanceolata Copel., Philipp. J. Sci., C 7: 64. 1912. Type: MALAYSIA. Sarawak: Bungo Range, Brooks 115(holotype: MICH).
Oleandra samoensis Gand., Bull. Soc. Bot. France 66: 306. 1919. Type. SAMOA. Upolu: Reinecke s.n. (not seen).
Oleandra colubrina var. *membranacea* Brause, Bot. Jahrb. Syst. 56: 119. 1921. Type: PHILIPPINES. Luzon: Mt. Banajao, Whitford 999 (holotype: B, not seen).
Oleandra parksii Copel., Bull. Bernice P. Bishop Mus. 59: 86. 1929. Type: FRENCH POLYNESIA. Fiji: Parks 20759(holotype: BISH? not seen; isotypes: BM*, MICH, US*).
Oleandra platybasis Copel., Bull. Bernice P. Bishop Mus. 59 86. 1929. Type: FRENCH POLYNESIA. Fiji: Gillespie 3249 (holotype: BISH? not seen; isotypes: MICH, NY*, UC*).
Oleandra angusta Copel., J. Arnold Arbor. 12 48. 1931. Type: SOLOMON ISLANDS. Vanikoro: Kajewski 5371 (holotype: A? not seen; isotypes: US*; UC*).
Oleandra maquilingensis Copel., Philipp. J. Sci. 46: 217. 1931. M.G.Price, Philipp. Agric. 57: 42. 1974. Zamora & Co, Guide to Philippine flora and fauna 2: 145. 1988. Amoroso & Pava, Philipp. J. Sci. 120: 423-437. 1991. Type. PHILIPPINES. Luzon: Matthew s.n.(lectotype: MICH 1210440, here selected, see discussion).
Oleandra archboldii Copel., Philipp. J. Sci. 73: 346. 1940. Type: PAPUA NEW GUINEA. Brass 13002 (holotype: MICH*).
Oleandra subdimorpha Copel., J. Arnold Arbor. 24: 441. 1943. Type: PAPUA NEW GUINEA. Brass 6886 (holotype: GH, not seen, isotypes: BM*, MICH*).
Oleandra christopherseni C. Chr, Bull. Bernice P. Bishop Mus. 177 47. 1943. Type: SAMOA. Christophersen 126 (holotype: BISH; isotype: BO). *Oleandra clemensiae* Copel., Philipp. J. Sci. 81 12. 1952. Copel., Fern Flora of the Philippines: 182. 1958. Type: PHILIPPINES. Clemens 16494 (holotype: MICH; isotype: UC*).
Oleandra herrei Copel., Philipp. J. Sci. 81 12. 1952. Copel., Fern Flora of the Philippines: 182. 1958. Type: PHILIPPINES. Herre s.n.(holotype UC*).
Oleandra malasianum Ghosh, J. Bombay Nat. Hist. Soc. 80: 630. 1984. Type. MALAYSIA. Penang: : Cantor, Wallich 2235 (CAL, not seen, ill. in Ghosh 1984).

#### Description.

*Rhizome* with main stems creeping or ascending, 3–8 mm thick, white waxy in the older parts, creeping parts sparsely rooting, branches often in opposite pairs, ascending parts rootless, at base propped up by downwards directed branches, ultimately aerial, erect or pendent; branches single or in opposite pairs, mostly directly above a frond cluster, all parts in cross-section with a peripheral sclerified sheath and scattered sclerified strands, phyllopodia on creeping parts few, scattered, on aerial parts in more or less dense, often whorled clusters and branches, short to 15 mm long. *Scales* persistently covering the rhizome, peltate, 4–6.5 × 1–1.5 mm, appressed to squarrose, with dark center and lighter margin and acumen, margin ciliate. *Fronds* monomorphic or weakly dimorphic, stipe short to 3.5 cm long, without dark colouration; fertile lamina 12–43 × 0.5–4.5 cm, base gradually narrowed to narrowly truncate, then often somewhat lyrate, apex acuminate or to c. 2 cm caudate; sterile, if present, usually slightly shorter and wider, to 36 × 5.5 cm; texture thin-chartaceous, costa and lamina on lower surface without dark colouration, glabrous or with up to 2 mm long hairs, costa often with up to 2 mm pale to dark narrow scales. *Sori* in a single row close to the costa, or more scattered over a 2–5 mm wide zone close to or at a distance of up to 4 mm from the costa, indusium inconspicuous to distinct, to c 1.5 mm wide, glabrous to hairy. Sporangial stalk with glands below the sporangium. Spores with coarse confluent ridges, surface pustulose or with pointed excrescences, outer layer variably perforated.

#### Distribution.

India (Himalayas), China (Xizang), Malesian archipelago to Australia, Pacific Islands (Fiji, Samoa).

**Figure 12.**
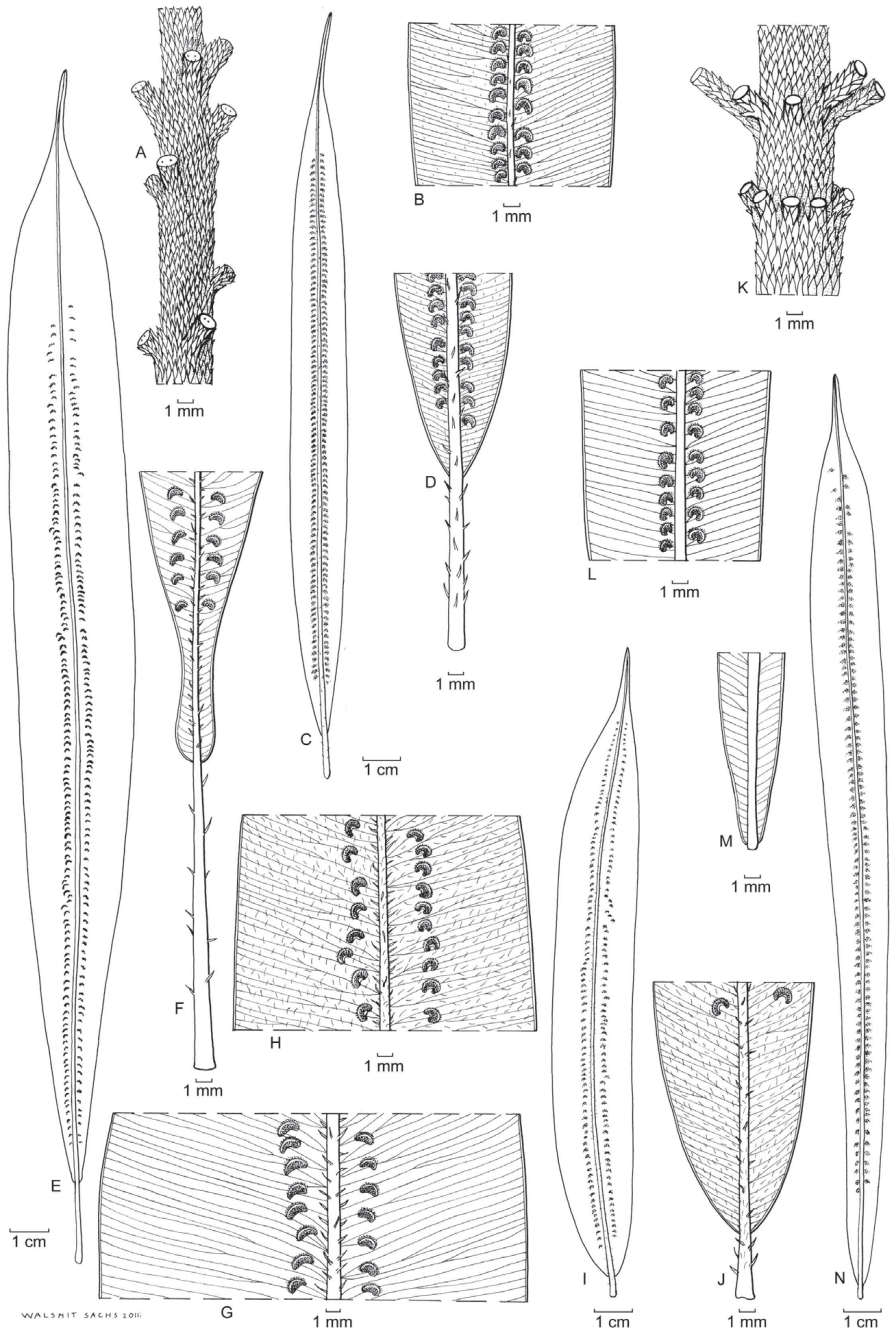
*Oleandra neriiformis*. **a** scattered phyllopodia on aerial stem **b** middle part of lamina with very closely costal sori and conspicuous indusia **c** lamina **d** lamina base with long stipe : *A.C. Smith 6104* (Fiji) **e** lamina **f** lyrate lamina base with long stipe **g** middle part of lamina, sori costal with conspicuous indusia: *Croft 66* (New Guinea) **h** middle part of lamina, sori costal with conspicuous indusia **i** lamina **j** cuneate lamina base with distinct stipe: *Van Balgooy 5223* (Java) **k** irregularly whorled phyllopodia on aerial stem **l** middle part of lamina with very closely costal sori and conspicuous indusia **m** narrowed lamina base with articulation close to the lamina base **n** lamina: *Chew W-L 94*2 (Peninsular Malaysia). All views of lamina showing abaxial surface; all specimens L.

#### Ecology.

Terrestrial or epiphytic, in various types of forests, in open places, often making up a significant part of summit or ridge scrub. Sea level to 2200 m.

#### Discussion. 

The epithet *neriformis* published by Cavanilles has to be corrected to “*neriiformis*” (ICBN 60.8). The epithet *neriifolia* used by Presl is either a mistake to be corrected or a superfluous *nom. nov*. and should not be used.

*Oleandra neriiformis* does not occur in Australia. The reference in Bell (1998) to *Oleandra neriiformis* refers indeed to *Oleandra musifolia* as suggested.

*Oleandra maquilingensis* is based on two sterile specimens that are considered to represent juvenile plants by Price (1974). Price selected, but did not publish, a lectotype from among the two, and we here follow that lectotypification. The identification of these sterile specimens with *Oleandra neriiformis* rather than *Oleandra cumingii* is somewhat conjectural, as is the identification of *Blechnum colubrinum* Blanco, for which no specimens at all are available.

*Aspidium phyllarthron* is one of the two species distinguished by Kunze (1848) among the specimens distributed as *Zollinger 1306*. In addition, Kunze cited *Zollinger 1957*. We have not found any specimens of *Zollinger 1957*, and from the several specimens that have been distributed as *Zollinger 1306*, we have selected one that fits the description of *Aspidium phyllarthron* and has a label “1306=1957”. Another specimen, labeled “*Zollinger 1306 a*, *Aspidium phyllarthron*” (L 0317412), does not fit the description and is *Oleandra musifolia*.

#### Variability.

With its wide-creeping and persistent rhizome, *Oleandra neriiformis* may form extensive and probably long-lived stands, which, especially when it is collected a number of times over a long period, may give the impression of the presence of a locally abundant species with a highly constant and distinct combination of characters. We expect this is at least partly the basis for the multitude of local species that have been described, and that we all include in *Oleandra neriiformis*. Another problem is posed by the occurrence of juvenile plants in which the rhizome is not characteristically developed, and which may have much more softly hairy fronds than well-developed plants, blurring the distinctions to *Oleandra musifolia* and *Oleandra cumingii*.

The following characters or character complexes, some of which have been used to distinguish species, are variable in particular:

1 Place of the stipe articulation. The phyllopodium may be distinctly longer than the very short stipe (fig. 12 k, n), or a distinctly elongated stipe may be present equal to or longer than the phyllopodium (fig. 12 a, d)

2 Length and density of lamina hairs. Although the presence or length of lamina hairs is usually highly variable, some forms have constantly and distinctly longer hairs.

3 Location of soral zone. Sori may be located in a narrow zone close to the costa (fig. 12 b, n), or in a more irregular zone at some distance from the costa (fig. 11 b, m).

4 Indusium. The presence of an indusium is rarely constant over an area. It may vary from distinct and often firm (fig. 11 h, 12 b) to inconspicuous (then often hairy) or absent (fig. 11 a, k).

5 Costal scales: Some forms have uniformly pale and flat costal scales, some have almost uniformly narrow, dark scales, and there are forms that vary in this character.

#### Geographic variation and local forms.

Over most of the distribution area, two forms can often easily be distinguished locally, on basis of the relative length of phyllopodium and stipe. Stipitate forms have short phyllopodia, elongated stipes (thus the articulation is positioned at the base of the phyllopodium/stipe), the lamina gradually narrowed towards the base, and sori relatively close to the costa (fig. 12 k–m). This corresponds to the type of *Oleandra neriiformis*. The other form is characterized by longer phyllopodia, stipes short or absent (thus the lamina appearing sessile with regard to the articulation, fig. 2 b), usually a truncate lamina base (although the lamina directly above the base may be strongly narrowed, the base is still suddenly contracted, and often somewhat lyrate, fig. 2 c) that is clearly set off against the stipe, and sori in a more variable position, in some cases almost at the margin (fig. 2b). Other characters, such as indument, or indusium are independently variable and often vary in a similar way across the two forms where they co-occur, thus giving rise to different characteristic character combination in different parts of the distribution area.

***Continental Asia*.** The few collections from continental Asia do not allow for an evaluation of the variability.

***Java, Sumatra*.** On Java and Sumatra, there is no distinction between stipitate and sessile forms, as both stipe and phyllopodium lengths are strongly variable and extremes are not sharply separated. In Backer and Posthumus (1939), all are taken together as *Oleandra neriiformis*. Description (see also fig. 12 h–j): Phyllopodia 2–6 mm, stipes 3–10 mm, lamina base narrowed, never lyrate, lamina variably hairy (mostly glabrous), costal scales usually few, medium dark; sori costal or at short distance from the costa, indusia distinct to firm, glabrous.

***Peninsular Malaysia, Southern Thailand*.** In collections from the Malay Peninsula the difference between stipitate and sessile forms is associated with differences in hairiness of the lamina and position of the sori. Holttum (1968b) also distinguishes these two forms, on basis of the same characters, but notes that that they are not sharply distinct, nor ecologically sharply separated, although he cites a difference in elevational specificity. The scant label data indicate no differences in specificity for epiphytism vs terrestrial, or for elevation. Stipitate form description: Phyllopodia very short, stipes elongated, c. 5–10 mm long, lamina glabrous, occasionally hairy, margin glabrous or very nearly so, sori subcostal in an irregular row. Sessile form description (see also fig. 12 k–n): Phyllopodia elongated, 1–6 mm long, stipes mostly very short, sometimes to 3 mm long, lamina hairy, sori strictly costal (rarely to 1 mm from costa).

***Borneo*.** On Borneo, the two forms also differ in hairiness of lamina, but not in the position of the sori, which is usually more or less closely costal. Here, the stipitate form is almost exclusively reported as epiphyte, the other form as terrestrial. Both forms tend to have narrower, darker costa-scales than in other areas. Completely glabrous forms such as are most common on Java and Sumatra are not found on Borneo. The sessile form has been described as *Oleandra oblanceolata* Copel. Stipitate form description: Phyllopodia up to 6 mm long, stipe 7–25 mm, lamina at base gradually narrowed, usually hairy with relatively long hairs (rarely glabrous), costal scales more or less frequent, mostly brown. Almost exclusively reported as epiphyte. Sessile form description (see also fig. 11 g–j): Phyllopodia 2–8 mm long, stipes at most 1 mm, lamina at base truncate, often ± lyrate, lamina mostly glabrous, sometimes short-hairy, costal scales not frequent, dark, narrow. Mostly reported as terrestrial.

***Philippines*.** On the Philippines, distinctly stipitate forms represent a small minority of all collections (e.g., *Cuming 94, Soejarto 8874, PNH 3862, 8710*), but include the type of *Oleandra neriiformis* (Christensen 1937). The two forms here do not show any difference in degree of hairiness and position of the sori, but share a distinctly hairy lamina and more copious, pale, flat rachis-scales than the forms in other areas.

Copeland (1958) distinguishes *Oleandra neriiformis* from the short-stipitate form, and within the latter a number of species, based on details of indument: *Oleandra herrei*, with paleate costa, *Oleandra colubrina*, with setose costa and lamina, *Oleandra nitida*, with setose costa and glabrescent lamina. We find that although the density of costal scales varies strongly (it seems to be negatively and weakly correlated with the density of setae), scales can be found on all specimens, and the density of hairs on the lamina varies strongly. We see no basis on which these characters could lead to the distinction of clear groups.

Stipitate form description: Phyllopodia to 2 mm, stipes to c. 7 mm, lamina and margin hairy with highly variable density, costa mostly with many pale scales, sori not closely costal to medial or submarginal, indusia glabrous, often small, indistinct. Sessile form description (fig. 11 k–m): Phyllopodia 5–15 mm, stipes short, up to 1 mm long, otherwise similar.

***Celebes, Moluccas.*** In eastern Malesia south of the Philippines, two forms co-occur on Celebes and the Moluccas, while the stipitate form extends to the Solomon islands and Vanuatu. Both forms here have indusia that are frequently shortly setose.

Stipitate form description: Phyllopodia 1–5 mm, stipes 8–25 mm, lamina base gradually narrowed, costal scales pale except near base of lamina, lamina setose, lamina hairs relatively long, margin often distinctly fimbriate with hairs shorter or equal to those on lamina, sori costal, indusia firm, with wide sinus, usually glabrous.

Sessile form description: Phyllopodia 5–15 mm, stipes very short to occasionally 3 mm long, lamina base narrowed to ultimately cuneate, not distinctly lyrate, lamina indument often long, conspicuous on all veins, margin often distinctly fimbriate with hairs similar to these on the lamina, costal scales few or absent, dark, sori narrowly costal, indusium distinct and persistent but not firm, often with narrow sinus, sometimes setose.

Kato (1989) distinguishes the specimens from Ceram with a very small, setose indusium as *Oleandra cuspidata*, but this represents only the extreme state of variability.

***Fiji, Samoa*.** More eastwards in the Pacific Islands, there is no variability in the location of the articulation. All specimens from this area have elongated stipes, and can collectively be distinguished from the forms in other areas by the distinctly more lax clusters of fronds on the aerial stems (fig. 12 a), with the fronds mostly hardly clustered at all (a character in which they resemble *Oleandra pilosa* Hooker, from Tropical America). Regional differences show up mainly in the indument of the fronds. On Samoa, the lamina is less densely hairy than on Fiji, and the hairs on the lamina, when present, are distinctly shorter than those on the margin. Costa scales also differ, and are distinctly darker on Samoa than on Fiji, while sori are somewhat less strictly costal on Samoa.

Fiji form description (see also fig. 12 a–d): Phyllopodia 1–9 mm, stipes 5–35 mm, lamina base gradually narrowed, costal scales pale, lamina setose, lamina hairs 1–2 mm long, margin often distinctly fimbriate, hairs on margin shorter or at most equal to those on lamina, sori costal, indusia distinct, glabrous or setose,

Samoa form description: Phyllopodia 1–7 mm, stipes 2–28 mm, lamina base gradually narrowed, costal scales brown to dark, lamina glabrous or setose, with hairs to 0.5 mm long on the lamina, 1–2 mm long on the margin, sori costal or subcostal leaving a sterile zone of 0.5–3 mm wide, indusia distinct, glabrous or setose.

***New Guinea and surrounding islands*.** The stipitate form that extends eastwards from Celebes co-occurs, on the main island of New Guinea, with a sessile form that is often distinctly dimorphic and has sori often quite distant from the costa.

Stipitate form description (see also fig. 12 e–g): Phyllopodia to 4 mm, stipes 5–25 mm, lamina base gradually narrowed, costal scales pale except near base of lamina, lamina setose, with hairs 0.5–1.5 mm long, margin glabrous, sori costal or subcostal leaving a sterile zone of 0.5–2.0 mm wide, indusia firm, with wide sinus, usually glabrous.

Sessile form description (see also fig. 11 a–c): Phyllopodia to 4 mm, stipes very short or absent, articulation directly below the base of the lamina, fronds slightly but usually distinctly dimorphic, lamina narrowed to a truncate or somewhat lyrate base, costal scales mostly brown to dark, lamina glabrous or sometimes short setose, with hairs to 0.5 mm long, margin usually with a few scattered pale, acicular hairs especially near the base, sori medial or submarginal leaving a sterile zone of 0.5–7 mm wide, indusia small and inconspicuous in older sori, occasionally more distinct, often fringed with pale acicular hairs.

### 5. Oleandra sibbaldii

http://species-id.net/wiki/Oleandra_sibbaldii

Map 4

Oleandra sibbaldii Grev., Ann.and Mag.Nat.Hist.Ser.2 1: 327. 1848. Copel., Bull. Bernice P. Bishop Mus. 93: 61. 1932. Philipp. J. Sci. 73: 346. 1940. C.Chr., Bull. Bernice P. Bishop Mus. 177 47. 1943. Copel., Fern Flora of the Philippines: 183. 1958. Brownlie, The Pteridophyte Flora of Fiji: 157. 1977. M.Kato, J. Fac. Sci. Univ. Tokyo, Sect. 3, Bot. 14: 239. 1989. R.M.Tryon, Rhodora 102: 436, fig. 7. 2001. *Oleandra cumingii* var. *sibbaldii* Baker, Syn. Fil. (Hooker & Baker): 303. 1868. Type: FRENCH POLYNESIA. Tahiti: Sibbald s.n. (holotype: E, barcode E00417634, not seen).
Oleandra tricholepis Kunze, Kunze, Bot. Zeitung (Berlin) 9: 349. 1851. Type. INDONESIA. Bornea: Hupe s.n. (holotype: LZ, probably destroyed).
Oleandra whitmeei Baker, J. Bot. 5: 11. 1876. (“*whitmei*”). *Oleandra whitmeei* Copel., Polypod. Phil. Isl.: 49. 1905. Copel., Bull. Bernice P. Bishop Mus. 59 87. 1929. Type. SAMOA. Savai’i : Whitmee & Powell s.n. (holotype: K).
Oleandra gracilis Copel., Univ. Calif. Publ. Bot. 12: 397, pl. 52b. 1931. Copel.Philipp. J. Sci. 73: 347. 1940. Type: PAPUA NEW GUINEA. New Guinea: Keysser 74 (holotype: UC).
Oleandra crassipes Copel., Philipp. J. Sci. 73: 347, Pl. 2. 1940. Type: INDONESIA. New Guinea: Bernhard camp, Brass 12109 (holotype: UC; isotypes: BO, L).

#### Description. 

*Rhizome* long-creeping or pendulous, sparsely branching, 1.5–4.5 mm thick, very strongly white waxy, in cross-section without or with few scattered sclerified strands, phyllopodia scattered, (0.3–)0.8–4.0(–5.7) cm long, roots with distinct unbranched parts. *Scales* deciduous, exposing the rhizome in older parts, peltate, (3–)5–15(–18) × (0.3–)0.4–0.9(–1.2) mm, usually widest above the attachment, squarrose, reddish brown, margin entire or more or less densely set with sessile glands, acumen attenuated, long filiform apex. *Fronds* monomorphic; stipe (0.3–)0.5–3.5(–4.5) cm long, often with dark coloration on abaxial side; lamina to (4.5–)15–40(–58) × 1–4(–6) cm, widest in middle part, base attenuate to cuneate, sometimes rounded, rarely truncate, sometimes asymmetric, apex acuminate or up to 3 cm caudate, texture membranous to coriaceous, both sides sparsely to densely set with catenate hairs to c. 0.5 mm long; costa on lower surface often with dark-coloration, on both surfaces with scales, scales on lower surface often abundant, to 6(–11) × 1(–1.2) mm, pale to dark brown, on upper surface scarce, inconspicuous. *Sori* inframedial, leaving a distinct 1–4 mm wide sterile zone between costa and soral zone, sometimes as much as 12 mm from costa, indusium firm, to 1 mm wide, hairy. Sporangial stalk with glands below the sporangium. Spores with an irregular mesh-like network of up to 10 µm high folds, finely papillose with spinules up to ca. 4 × 1 μm, perispore baculate, outer layer much perforated.

#### Distribution.

Eastern Malesia to Pacific Islands. Malaysia: Sabah, Sarawak, Philippines: Mindanao; Indonesia: Sulawesi; Moluccas, Papua; Papua New Guinea; Solomon islands; Vanuatu; Tahiti and Marquesas: Hiva Oa, Nuku Hiva, Tahuata, Ua Huka and Ua Pou; Western Samoa: Savaii; Fiji.

**Map 4.**
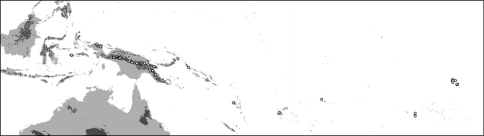
Distribution of *Oleandra sibbaldii*.

#### Ecology.

Epiphytic, epilithic, or less commonly terrestrial (most often at elevations over 1500m), commonly collected from mossy forests, climbing or sprawling among bryophytes and other epiphytes, or pendulous from mossy tree branches, to 600 m (Marquesas and Tahiti); or from 1000 to 3000 m (elsewhere).

#### Discussion.

*Oleandra sibbaldii* is common on New Guinea, but appears to be much sparser towards the periphery of the distribution area.

The dark colour on the abaxial surface of the costa is often very conspicuous, extending on the stipe, thus rendering the stipe conspicuously bicolorous.

### 6. Oleandra undulata

http://species-id.net/wiki/Oleandra_undulata

Map 2

Oleandra undulata Ching, Lingnan Sci. J. 12: 565. 1933. Holttum, Rev. Fl. Mal. 2: 384. 1954. Ching, Fl. Reipubl. Popularis Sin. 2: 322. 1959. X.C.Zhang, Ching Mem. Vol.: 92. 1999. R.M.Tryon, Rhodora 102: 346, fig. 6. 2001. *Polypodium undulatum* Willd., Sp. Pl., ed. 4 [Willdenow] 5: 155. 1810. Type: INDIA. Tranquebar: Klein 887 (holotype: B - Willdenow 19616 -01 0).
Oleandra pubescens Copel., Univ. Calif. Publ. Bot. 12: 397, Pl. 52A. 1931. Type: THAILAND. Eryl Smith 1072 (holotype: UC*).

#### Description. 

*Rhizome* short-creeping, little branching, 5–6 mm thick (3–5 when dry), not white waxy, in cross-section with many scattered sclerified strands; phyllopodia irregularly scattered, close together or distant, 2–8 cm long, roots scattered, without distinct unbranched parts. *Scales* persistently covering the rhizome, peltate, 3–5 ×1–1.6 mm, slightly spreading, acumen brown, with sparsely ciliate margin, apex short, wide. *Fronds* monomorphic; stipe 3–15 cm long, without dark coloration, articulation at 1/5–1/2 from base; lamina to 60 × 1–5.5 cm, widest in middle part, base truncate or cuneate to gradually narrowed, apex acute to narrow-acuminate, texture herbaceous, upper surface glabrous or sparsely, lower surface more densely set with usually acicular, sometimes slightly catenate hairs to c. 0.5–1.0 mm long; costa on lower surface without dark coloration, without scales. *Sori* close to costa or leaving a distinct 1–6 mm wide sterile zone between costa and soral zone, indusium firm, to 2 mm wide, hairy. Sporangial stalk without glands below the sporangium. Spores finely papillose and coarsely ridged, perispore baculate, outer layer not perforated.

#### Distribution.

Burma, Laos, Thailand, China: Yunnan. Mostly below 1000 m.

#### Ecology.

In open or deciduous forests, often disturbed; terrestrial or epilithic, rhizome subterraneous, on rocks or in crevices, mostly on granite.

#### Discussion.

*Oleandra undulata* can be difficult to distinguish from *Oleandra cumingii*. In addition to the differences listed under that species, subterraneous growth of the rhizome may be characteristic for *Oleandra undulata*, but field observations are lacking for many specimens.

### 7. Oleandra vulpina

http://species-id.net/wiki/Oleandra_vulpina

Map 5

Oleandra vulpina C.Chr., Dansk Bot. Ark. 9 68. 1937. Type. PAPUA NEW GUINEA. New Guinea: Ledermann 7652 (holotype: BM).

#### Description. 

*Rhizome* long-creeping, ca. 3 mm thick, not white waxy, sparsely branching, roots with unbranched parts; in cross-section with weakly developed sclerified shealth and few scattered sclerenchyma strands, phyllopodia scattered, 3–6 cm distant, 6–7 mm long. *Scales* scattered, not covering the rhizome, peltate, to 5 × 0.5 mm, appressed at the base, with a narrow squarrose acumen, dark brown near attachment, lighter towards margin, margin densely set with glands and multicellular hairs terminating in a gland. *Fronds* monomorphic; stipes with scales as the rhizome but less dense and with short fine glandular hairs; stipe 2–3 mm long, without dark coloration on abaxial side, bearing short fine glandular hairs; lamina linear-lanceolate, 17.5—20 × 3–4 cm wide, base cuneate, apex short caudate, tips up to 1 cm long, margin undulate, weakly cartilaginous, texture papyraceous, all parts with to 0.5 mm long catenate glandular hairs, or with longer, to 1.5 mm, acicular hairs; costa abaxially without dark coloration, with hairs like the stipe and with small scales; veins terminating in a weakly developed hydathode before the margin. Sporangial stalk with glands below the sporangium. *Sori* in an irregular row 2.5–6 mm from the costa, indusium round-reniform, c. 0.5 mm across, fugacious at very early stage. *Spores* absent.

#### Distribution.

New Guinea, at 975 m.

**Map 5.**
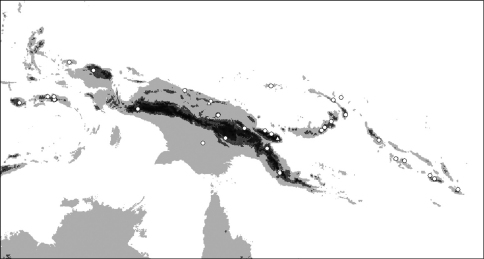
Distribution of *Oleandra vulpina* (triangles), *Oleandra werneri* (circles).

#### Ecology.

Scandent on trunk of Sago palm in garden, or in forest.

#### Vernacular name.

Taingelem (Wapi language, Miwaute)

#### Discussion.

*Oleandra vulpina* is distinct from *Oleandra sibbaldii* in the rhizome and costa scales. Rhizome scales are darker in colour with pale-coloured margin, long subulate apices, and margin strongly ciliate with glandular hairs. A similar glandular scale indument is found in the American *Oleandra articulata* (Sw). C. Presl, but not in the other Southeast Asian species. The costa scales beneath are small and inconspicuous, narrowly lanceolate. In addition, the rhizome of *Oleandra vulpina* is not glaucous. The indusia are very small and shrivel at a very young stage but are distinct when present and bear numerous setose hairs.

### 8. Oleandra wallichii

http://species-id.net/wiki/Oleandra_wallichii

Map 2

Oleandra wallichii C.Presl, Tent. Pterid.: 78. 1836. Fée, Mém. Foug., 5. Gen. Filic.: 304. 1852. Hook., Sp. Fil.: 158. 1862. Ching, Fl. Reipubl. Popularis Sin. 2: 321. 1959. Shieh, DeVol & Kuo in Huang, Fl. Taiwan, ed. 2.: 203, Pl. 83. 1994. X.C.Zhang, Ching Mem. Vol.: 94. 1999. R.M.Tryon, Rhodora 102: 434, fig. 5. 2001. *Aspidium wallichii* Hook., Exot. Fl.: Pl. 5. 1823. Pl. *Neuronia asplenioides* D.Don, Prodr. Fl. Nepal.: 7. 1825., nom. illeg. Type: NEPAL.Wallich s.n. (holotype K; isotypes: BM, PE).
Oleandra wallichii var. lepidota Christ, Bull. Acad. Int. Geogr. Bot. 15: 140. 1906. Type: CHINA. Western China: Wilson 5246 (holotype P; isotype: K).

#### Description. 

*Rhizome* creeping, 3–4 mm thick (2–3 when dry), white waxy in the older parts, with long, leafless parts alternating with more or less dense clusters of very short phyllopodia, usually less than 2 (–5) mm high, of which usually only 1–2 bear fronds at the same time, branches usually in opposite pairs; in cross-section with weak sclerenchyma sheath and without sclerified strands, roots scattered, usually with long unbranched parts. Scales persistently covering the rhizome, peltate, 3–7 ×1–1.3 (–1.5) mm, base appressed, with dark center and lighter margin, acumen brown, usually recurved, with ciliate margin, apex twisted and with frizzly cilia. *Fronds* monomorphic; stipe 1–5 cm long, with dark coloration on abaxial side often distinctly bicolorous, with catenate hairs; lamina 13–45 × 2–4.5 cm, base truncate to rounded, apex often abruptly caudate, texture thin-herbaceous, translucent, both surfaces densely set with catenate hairs 0.5–1 mm long; costa on lower surface in basal half of lamina with dark coloration, with copious 2–4 mm long pale scales. *Sori* close to costa, indusium thin, to 1 mm wide, glabrous or hairy. Sporangial stalk without glands below the sporangium. Spores echinate and ridged, perispore solid.

#### Distribution.

Himalayas to Northern Thailand, Yunnan and Taiwan, 1600 to 3600 m. India, Nepal, Bhutan, China (Yunnan, Taiwan), Thailand.

#### Ecology. 

Mostly epiphytic, on mossy trunks, also on cliff faces or boulders.

#### Discussion.

*Oleandra wallichii* differs from *Oleandra undulata* in the more wide-creeping rhizome that is often distinctly glaucous beneath and between the scales, without sclerenchyma strands; the often conspicuously bicolorous stipe, lamina with usually very distinctly apiculate apex and costa with frequent scales, sori constantly closely costal, with small indusia.

### 9. Oleandra werneri

http://species-id.net/wiki/Oleandra_werneri

Map 5

Oleandra werneri Rosenst., Repert. Spec. Nov. Regni Veg. 5: 40. 1908. Copel., Philipp. J. Sci. 73: 347. 1940. R.M.Tryon, Rhodora 102: 433, fig. 3. 2001. Type. INDONESIA. New Guinea: Werner 12(lectotype: L, “Rosenstock 28”, here selected; isotype: B).
Oleandra dimorpha Copel., Philipp. J. Sci. 60: 111. 1936. M.Kato, J. Fac. Sci. Univ. Tokyo, Sect. 3, Bot. 14: 239. 1989. Type. SOLOMON ISLANDS. San Christoval: Brass 2916 (holotype: MICH).

#### Description.

*Rhizome* with main stems creeping, scrambling or pendent, 2–3 mm thick, white waxy in the older parts, sparsely rooting, mostly terete when dry, bearing scattered short, usually less than 5 mm long phyllopodia, phyllopodia on aerial parts more closely together but not densely clustered; all parts in cross section with a peripheral sclerified sheath and scattered sclerified strands. *Scales* covering the rhizome, peltate, 3–4 × 0.5–1 mm, somewhat squarrose, with dark center and lighter margin and acumen, margin ciliate especially when young. *Fronds* strongly dimorphic, stipe 0.5–2.5 cm, without dark colouration, especially upwards with up to 2 mm long hairs; lamina chartaceous; fertile 27–56 × 0.5–1 (–1.7) cm, base and apex narrow, sterile 17–30 × 2.2–5.7 cm, base mostly cuneate, apex distinctly 2–3.5 cm caudate; costa and lamina on lower surface glabrous or with up to 2 mm long hairs, costa without dark colouration, like the stipe with up to 2 mm long brown scales. *Sori* in a single row on both sides of the costa, indusium firm, 1–3 mm wide, glabrous. Sporangial stalk with glands below the sporangium. Spores with coarse confluent ridges, areolae with short pointed excrescences, perispore hollow, with internal baculae, outer layer not or hardly perforated.

#### Distribution.

Indonesia (Maluku, Papua); Papua New Guinea; Vanuatu.

#### Ecology.

Commonly epiphytic, on trunks or in crowns, less often terrestrial or on rocks, erect, scrambling or pendent, in various types of forests, most frequently in montane or mossy forests, on ridges, up to c. 2000 m.

#### Discussion.

As in *Oleandra neriiformis*, rhizome morphology is probably more complicated than can be inferred from the mostly aerial unbranching parts making up most of the collected material.

Hairiness is very variable, and while there is no sharp distinction between hairy and glabrous forms, it is noteworthy that hairy forms tend to occur at especially the Western extreme of the distribution area, with less hairy forms near the Eastern extreme and glabrous forms mostly on the mainland of New Guinea.

## Supplementary Material

XML Treatment for Oleandra

XML Treatment for Oleandra coriacea

XML Treatment for Oleandra cumingii

XML Treatment for Oleandra musifolia

XML Treatment for Oleandra neriiformis

XML Treatment for Oleandra sibbaldii

XML Treatment for Oleandra undulata

XML Treatment for Oleandra vulpina

XML Treatment for Oleandra wallichii

XML Treatment for Oleandra werneri

## Appendix 1

### Identification list

*Oleandra coriacea*: 1

*Oleandra cumingii*: 2

*Oleandra musifolia*: 3

*Oleandra neriiformis*: 4

*Oleandra sibbaldii*: 5

*Oleandra undulata*: 6

*Oleandra vulpina*: 7

*Oleandra wallichii*: 8

*Oleandra werneri*: 9

Abbe, L.B.; Abbe, E.C. 9650 : 4; 9805 : 4; Abdul Samat, A. 118 : 4; Adelbert, A.G.L. 128 : 4; 252 : 4; 490 : 4; Aet; Idjan 324 : 4; Ajoeb 265 : 4; Allen, B.M. 1427 : 4; 1779 : 4; Alston, A.H.G. 16962 : 4; Anderson, J.A.R. S 18570 : 4; Andrews, S.B.; Stocker, G. 283 : 3; Ashton, P.S. 242 : 4; 424 : 1.

Backer, C.A. 12576 : 3; 23016 : 4; 25877 : 4; 36909 : 3; Bakhuizen v.d. Brink, R.C. 1539 : 4; 2777 : 4; 3208 : 4; 4482 : 4; 7677 : 4; Balgooy, M.M.J. van 1887 : 5; 5037 : 4; 5223 : 4; Balgooy, M.M.J. van; Wiriadinata, H. 2865 : 4; Bamler, G. 31 : 4; Bamler, M.G. ROS 132 : 4; Banoc, L.M. 3 : 6; 58 : 6; Banying ak Nyudong S 17211 : 4; S 19419 : 4; Barcelona, J.F. 2040 : 4; Barcelona, J.F.; Busemeyer, D.T. 717 : 4; Barcelona, J.F.; Busemeyer, D.T.; Ippoli, A. 575 : 4; 622 : 4; Bartlett, H.H. 15759 : 4; Beaman, J.H. 6957 : 4; 8027 : 4; 9605 : 4; 9921 : 4; 10332 : 4; Beusekom, C.F. van; Beusekom, R.J. van 1542 : 3; Beusekom, C.F. van; Charoenphol, C. 1690 : 3; Beusekom, C.F. van; et al. 4517 : 3; 4526 a: 3; 4813 : 6; Beusekom, C.F. van; Phengkhlai, C. 2431 : 8; Bor, S. 767 : 8; Borssum Waalkes, J. van 1290 : 4; 2824 : 4; Braithwaite, A.F. 4216 : 9; R.S.N.H. 2370 : 5; RSNH 2087 : 4; RSNH 2110 : 4; RSNH 2436 : 4; RSS 4045 : 9; RSS 4174 : 4; RSS 4471 : 9; RSS 4676 : 5; Brass, L.J. 2916 : 9; 3032 : 4; 3341 : 4; 3893 : 4; 7115 : 4; 11266 : 5; 11870 : 4; 12109 : 5; 12158 : 5; 12841 : 9; 12842 : 4; 13002 : 4; 13214 : 9; 13215 : 4; 13323 : 4; 23016 : 4; 23174 : 5; 24907 : 4; 25803 : 4; 25805 : 4; 26074 : 4; 26087 : 4; 29706 : 5; 31944 : 4; 32064 : 9; Britton, B.B. 326 : 4; Brooke, W.M.A. 8590 : 4; Brooks, C.J. 115 : 4; 263 s: 4; Brown, E.D.W. 980 A: 5; 980 B: 5; 980 C: 5; 980 D: 5; 980 E: 5; Brownlie, G. 1717 : 4; Bünnemeijer, H.A.B. 675 : 4; 3875 : 4; 4127 : 4; 4238 : 4; 5494 : 4; 8969 : 4; 11481 : 3; 12161 : 3; Burkill, I.H. 12904 : 4; SFN 8497 : 4; Buwalda, P. 3640 : 4; 5171 : 4; 6622 : 4; 8063 : 4.

Caerlan; Sageed; Romero PPI 13079 : 4; Carr, C.E. 13428 : 4; 13552 : 5; 13981 : 9; 14419 : 5; 14831 : 9; Carrick, J. JC 102 : 4; Chai, P. S 37561 : 4; S 39456 : 4; Chao Yu Zhang 21441 : 8; Charoenphol, C. 4241 : 3; Cheesman, L.E. 128 : 4; 1199 : 9; Chen Nian-qu 40930 : 2; Cheng Shu-Zhi; Li Bosheng 1015 : 8; 5250 : 4 Chew, W.L. 942 : 4; 1273 : 4; Chew, W.L.; Corner, E.J.H.; Stainton, A. 298 : 4; 1461 : 4; Chin, S.W. 28 : 4; Ching, R.C. 25432 : 8; Christophersen, E. 15 : 4; 125 : 4; 520 : 4; 826 : 5; 866 : 4; 2138 : 5; 3095 : 5; Christophersen, E.; Hume, E.P. 2305 : 4; Chun, N. 40930 : 2; Cicuzza, D. 178 : 4; Clarke, C.B. 42231 A: 6; Clemens, J. 421 : 4; 4534 : 9; 32552 : 5; 32871 : 5; Clemens, J.; Clemens, M.S. 27210 : 4; 28337 : 4; 28763 : 4; 30705 : 4; 30902 : 4; Clemens, M.S. 9114 : 5; 11053 : 4; 16494 : 4; 41009 : 5; Clunie, N.M.U. LAE 63276 : 9; LAE 63380 : 9; LAE 63399 : 4; Co, L. 3197 : 4; Coert, J.H. 644 : 4; 1497 : 4; Collecteur de Darjeeling 109 : 8; Coode, M.J.E. 6498 : 4; NGF 32846 : 4; Coode, M.J.E.; et al. 7548 : 4; Copeland, E.B. 137 : 4; 1381 : 4; 1474 : 4; 1766 : 4; 1804 : 2; 2016 : 4; ppe 56 : 4; Cox, P.A. 224 : 4; 345 : 4; 838 : 4; Craven, L.A. 1123 : 5; 1269 : 5; Craven, L.A.; Schodde, R. 133 : 4; 287 : 9; Croft, J.R. 34 : 4; 66 : 4; 224 : 9; 817 : 4; 821 : 4; 1223 : 4; 1897 : 9; 1987 : 9; Croft 11 : 5; Croft 875 : 5; Croft 1508 : 5; LAE 65781 : 5; LAE 65822 : 5; LAE 68337 : 5; LAE 68911 : 4; LAE 71012 : 4; LAE 71112 : 4; Croft, J.R.; Katik, P. NGF 14933 : 4; Croft, J.R.; Lelean, Y. LAE 65642 : 4; Croxall, J.P. 6016 : 5; Cuming, H. 60 : 2; 94 : 4; Curry, P. 1644 : 4; Curtis, C. 160 : 4.

Damas, K. LAE 58855 : 4; Danser, B.H. 5971 : 4; Darbyshire, P.J. 226 : 7; 325 : 9; Darnaedi, D. 1554 : 5; 2362 : 4; 2725 : 3; Davidse, G.; Sumithraarachchi, D.B. 7965 : 3; Davis, A.P. 464 : 4; 814 : 5; 814 : 5; Degener, O. 14279 : 4; Docters van Leeuwen, W.M. 10942 : 4; Dodd, J. E 10 : 9; Dransfield, S. 976 : 4.

Ecology Highland Group 14053 : 8; Edaño, G.E. 26 : 4; 71 : 4; 303 : 4; 637 : 4; 647 : 4; 796 : 4; 806 : 4; 1540 : 4; 2596 : 4; 5922 : 4; 6260 : 4; 8031 : 4; pnh 17267 : 4; Edwards, P.J. 724 : 1; 1988 : 4; 2141 : 4; Elbert, J. 1650 : 3; Elmer, A.D.E. 7940 : 4; 9069 : 4; 9959 : 4; 10908 : 4; 11451 : 5; 14140 : 4; 14149 : 4; 17694 : 4; 17964 : 4; Endert, F.H. 3030 : 4; 4244 : 4; Evans, J.H.N.; Gordon, W. 998 : 2; Eyma, P.J. 1664 : 5; 4484 : 9; 4726 : 4.

Fallen, M.E. 437 : 5; Faurie, U. 611 : 8; Florence, J. 3634 : 5; 4336 : 5; 6780 : 5; 7498 : 5; 9623 : 5; 9770 : 5; Forbes, H.O. 662 : 3; 884 : 3; 3482 : 2; Foreman, D.B. LAE 59150 : 9; NGF 45759 : 4; forest production group 84422 : 2; Forrest, G. 11799 : 8; 18581 : 8; 24238 : 8; 26691 : 8; Fosberg, F.R. 62670 : 5; Foxworthy, F.W. 72 : 4; 77 : 4; 359 : 4; Fu Guoxun 415 : 8; Fuchs, H.P.; Collenette, S.H. 21671 : 4.

Gaerlan, F.J.M.; Sagcal, E.; Romero PPI 10874 : 4; PPI 13079 : 4; Gagné, B.H. 1113 : 5; 1580 : 5; BHG 2318 : 5; Garber, D.W. 724 : 4; 1046 : 4; Gawi, M. 11 : 5; Gebo, A. 1619 : 4; Geesink, R.; Santisuk, T. 5384 : 2; Ghose, G. 11 : 3; Gideon, O. LAE 57504 : 4; Gideon, O.; Silu, J. LAE 76918 : 4; Gillespie, J.W. 2748 : 4; 3249 : 4; 3815 : 4; 4110 : 5; Gillett, G.W. 2174 : 5; Gong Wu Su 479 : 8; Graeffe 1078 : 4; Grant, M.L. 3704 : 5; 4095 : 5; Gravendeel, B. 593 : 4; Grey; Wilson, E.H.; Phillips 289 : 3; Gurung, V.L. 55 : 2; Gwynne Vaughan, D. 431 : 4.

Haas, J.H. de 2622 : 8; Hallier, H. 418 : 4; 1699 : 4; 3297 : 4; Hansen, B.; Smitinand, T. 11846 : 2; Hansen, C. 1128 : 4; Hardeveld, C. van 318 : 3; Hartley, T.G. 11417 : 4; Harvey, H.D. 1668 : 4; Hassan Flora Project 579 : 3; 692 : 3; 887 : 3; Henderson, M.R. 11197 : 4; 17879 : 4; Hennipman, E. 3092 : 3; 3334 : 6; 3334 A: 2; 3334 B: 6; 3413 : 8; 3568 : 3; 3655 : 3; 5370 : 5; 5430 : 5; 5518 : 4; 6158 : 4; Henry, A. 9484C : 2; 9484 : 8; Hirano, M.; Hotta, M. 1522 : 4; Hirschland, J.G. 2 : 4; Hochreutiner, B.P.G. 1755 : 4; 3326 : 4; Hodel, D.R. 1373 : 5; Höft, R. 2260 : 5; 2733 : 5; 2953 : 5; 3659 : 5; Holstvoogd, C. 471 : 4; 472 : 3; 847 : 3; 848 : 3; Holttum, R.E. 1668 : 4; 20580 : 4; 31319 : 4; N.G.F 40181 : 5; SFN 10686 : 4; SFN 11403 : 4; SFN 14852 : 2; SFN 25723 : 4; SFN 39209 : 3; Hoover, W.S. 434 : 9; Hortus, Bogor 57 : 4; 177 : 4; Hosokawa, Takahide 8677 : 4; Hotta, M. 14797 : 1; Hou, D. 213 : 4; Hovenkamp, P.H. 527 : 4; 05 05 : 4; Huang, T.C.; Kao, M.T. 1730 : 8; Hume, H.L. 7174 : 4.

Investigation team in western Yunnan 11416 : 8; Iwatsuki, K. 484 : 8; 531 : 3; B 1870 : 4; B 2495 : 4; B 2505 : 4; C 921 : 9; P 597 : 3; Iwatsuki, K.; Fukuoka, N. T 3424 : 6.

Jaag, O. 1110 a: 3; 1170 : 3; 1960 : 4; Jacobs, M. 7948 : 4; Jacobson, E. 2549 : 4; Jermy, A.C. 3488 : 5; 13677 : 4; 14420 : 1; Jiang Xinglin 35239 : 8; John, H.; St 17025 : 5; Johns, R.J. 7369 : 4; 7986 : 5; 9023 : 5; 9091 : 5; 10303 : 4; 10329 : 5; Jones, W.B. 1798 : 5.

Kadim bin Tassim K 491 : 2; Kajewski, S.F. 874 : 4; 2691 : 4; Kalkman, C. 4005 : 4; 4379 : 5; 4469 : 5; Kathmandu Expedition KE 1067 : 8; Katik, P. LAE 56318 : 4; Kato, M. 822 : 9; B 3674 : 4; B 6042 : 4; B 7667 : 4; B 7901 : 4; B 9511 : 1; B 9569 : 1; B 9813 : 1; B 10920 : 1; B 11131 : 4; C 1278 : 9; C 1365 : 4; C 1729 : 4; C 1945 : 4; C 2907 : 4; C 4121 : 4; C 5058 : 4; C 7412 : 9; C 7480 : 5; C 11656 : 4; C 12846 : 5; C 12965 : 4; C 13351 : 4; C 14279 : 4; C 14361 : 5; C 14446 : 4; Kato, M.; Setoguchi, H.; Darnaedi, D. 1160 : 4; Kato, M.; Wiriadinata, H. B 4749 : 4; B 4924 : 4; B 5283 : 4; B 5931 : 4; Kaudern, W. 86 : 4; Keenan 1386 : 6; Kerr, A.F.G. 9363 : 6; 9645 : 3; Kessler, P.J.A. 2857 : 4; Keysser, C. II 74 : 5; Kinbag, F. 013 : 5; King’s collector 2424 : 4; Kog, P. 004 : 5; Koorders, S.H. 14942 B: 4; 19832 b: 3; 36678 B: 4; Koster, C. BW 13848 : 4; Kostermans, A.J.G.H. 2280 : 4; 5549 : 4; 6513 : 4; Kostermans, A.J.G.H.; Soegeng, W. 509 : 4; Kostermans, A.J.G.H.; Wirawan, N. 747 : 3; Kuhl, H.; Hasselt, J.C. van 7 : 4; Kurz, S. 203 : 3.

Lace, J.H. 4227 : 6; LAE 65715 : 4; Lai, J.; Enjah S 64162 : 4; Lam, H.J. 537 : 4; 1449 : 4; 1856 : 5; 1943 : 5; 6897 : 4; Lang Kai-Yong; Zhang Yongtian 3360 : 8; Larsen, K. 44 : 3; 964 : 3; 2689 : 6; 8936 : 6; 10678 : 3; 31861 : 6; Larsen, K.; Larsen, S.S. 30713 : 3; Lau S.K. 1490 : 2; 3108 : 2; 27368 : 2; Lavarack, P. NGF 31446 : 5; Lavarack, P.; Ridsdale, C.E. NGF 31444 : 9; LeBronnec, G. 809 : 5; Ledermann 7652 : 7; Leeuwenberg, A.J.M. 3 : 4; Li Bosheng 14023 : 8; Li Hua Hou 1009 : 2; Li Yang Yi; Li Bosheng 13537 : 8; Liu Bingrong 5064 : 2; 5065 : 2; Lorence, D.H. 6094 : 5; Lörzing, J.A. 728 : 3; 5202 : 4; 6890 : 4; 14050 : 4; Luang Winit-Wanadorn 47 : 6; Luang, Winit 1003 : 3; Ludlow, F. 6496 : 8; 6685 : 8; Ludlow, F.; Sherriff, G.; Hicks, J.H. 17008 : 8; 17416 : 8; 17499 : 8; 17536 : 8; 17540 : 8; 19563 : 8; 20909 : 8; Luerssen 3766 : 4; 9902 : 4.

M. K. Li 450 : 2; MacDaniels, L.H. 1531 : 5; Main 261 : 4; Mamit, J.D. S 34391 : 4; Mangen, J.M. 2227 : 4; Manickam, V.S.; Matthew, K.M. RHT 34242 : 3; Manner; Street 346 : 4; 592 : 4; Manseima, J. 011 : 5; Martellino, A.; Edaño, G.E. BS 35643 : 4; Maxwell, J.F. 85-194 : 4; 74 777 : 3; 74 907 : 6; 76 571 : 3; 78 167 : 4; 85 495 : 4; 87 227 : 2; 87 641 : 6; 89 858 : 6; 91 495 : 6; 92 716 : 6; 93 774 : 3; 93 1359 : 6; 94 1030 : 8; 94 1303 : 6; 95 919 : 6; 96 1265 : 3; 97 720 : 6; 97 1082 : 6; 98 636 : 6; McClure, F.A. 20061 : 2; McGregor, R.C. BS 10316 : 4; McKee, H.S. 3054 : 5; Meebold 7512 : 6; Mei Fung 4879 : 8; Meijer, W. 69 : 3; 905 : 4; 9473 : 4; 10341 : 3; Mendoza, D.R.; Convocar, P. 712 : 4; 748 : 4; Merrill, E.D. 624 : 4; 3238 : 4; 5937 : 4; 6682 : 2; Sp. Blan 490 : 4; Middleton, D.J.; Meng Monyrak 617 : 4; Millar N.G.F. 15745 : 5; Mogea, J.P.: Wilde, W.J.J.O de 3715 : 4; Mohd Haniff SFN 8019 : 4; Mohd Nur SFN 34358 : 4; Mohd Shah MS 656 : 4; MS 2803 : 4; Molesworth Allen, B. 4574 : 2; Moulton, J.C. SFN 6764 : 4; Moysey, L. 31884 : 4; Mumford, E.P. 472 : 5; Murata, G. J 890 : 4; T 16057 : 8.

Nakaike, K. 558 : 5; native collector 2222 : 4; SFN 4179 : 2; Neervoort, A.M. 253 : 4; Nitta, A. 15091 : 3; Normal School Students 12691 : 4; Norris, W. 856 : 4.

Oliver, R.L. 3109 : 5; Ooststroom, S.J. van 14090 : 3.

Pacific Entomological Survey 474 : 5; Ex 472 : 5; Palmer, W. 512 : 4; Palmer, W.; Bryant, O. 254 : 3; Parks, H.E. 20759 : 4; 70904 : 4; Parris, B.S. 7913 : 5; 10550 : 4; 11252 : 4; 11331 : 4; Parris, B.S.; Croxall, J.P. 5968 : 4; Parris, B.S.; Edwards, P.J. 10479 : 2; Parry Hance 1998 : 2; Pearce, K.G.; Serukit, D. S 95467 : 1; Perlman, S. 10118 : 5; Petrmitr, O. 164 : 6; 397 : 6; Phengkhlai, C. 283 : 2; 305 : 6; Phoon, S.N. FRI 53265 : 4; Pierre, L. 5713 : 4; Piggott, A.G. 2187 : 4; Pleyte, D.R. 343 : 4; Poilane, E. 4219 : 3; 9540 : 6; 12673 : 8; 15805 : 2; 23031 : 4; Polak, A.M. 931 : 4; Polak, E. 2048 : 4; Pooma, R. 4830 : 6; Pooma, R.; et al. 4373 : 4; Posthumus, O. 2429 : 4; 4058 : 4; Price, M.G. 1157 : 4; 2691 : 4; Price, M.G.; Hernaez, B.F. 701 : 4; Pulle, A.A. 113 : 4; 484 : 4; 596 : 4; 722 : 5; 1086 : 5; Pullen, R. 7738 : 4; 8069 : 4.

Qi Xinping Q 77 : 8; Qinghai-Tibet Team 4427 : 8; 4920 : 8; 8673 : 8; Qiu Yun 52907 : 8; Quayle, E.H. 61 : 5; 1145 : 5; 1258 : 5.

Raap, H. 218 : 4; 227 : 4; Rachmat 496 : 4; Ramos, M. 14881 : 4; Raynal, J. 17073 : 4; Raynal, J.; Schmid, R. RSNH 16123 : 4; Reinecke, F. 108 : 4; Reinecke, F.? 94 : 4; Reynoso; Garcia; Sagcal, E. PPI 14300 : 4; Ridley, H.N. 571 : 2; 5171 : 2; 5172 : 2; 7832 : 4; Riswan, S.; Afriastini, J.J. J 075 : 4; Robinson, H.C. 6024 : 4; Rock, J. 2026 : 6; Römer, L.S.A.M. von 770 : 4; Royen, P. van 3644 : 4; 3842 : 4; 3870 : 5; 7373 : 5; Royen, P. van; Sleumer, H.O. 5959 : 9; 6344 : 4; 7485 : 4; 7651 : 4; Rutten 1567 : 4; 2047 : 9; 2198 : 9; 2244 : 4.

S. Y. Dong 136 : 3; Sachet, M.-.H. 1021 : 5; Saigol, P. SAN 93067 : 4; Saldanha 17990 : 3; Sands, M.J.S. 6399 : 4; Sauveur; Sinke 2516 : 4; Schiffner, V. P 209 : 4; Schlechter, R. 16689 : 9; 18572 : 4; 19626 : 4; Schmutz, E. 59 : 3; 636 : 3; 2282 : 3; 6086 : 2; F 24 : 2; Schnell, R. 10176 : 3; Schwartz, A. 2448 : 4; 2795 : 3; 2822 : 4; Scortechini, B. 394 : 4; Seimund, E. 236 : 4; Setchell, W.A. 398 : 4; Shimizu, T. M 13244 : 4; T 8959 : 2; T 11457 : 3; T 22791 : 2; Shui Yumin 3730 : 8; Sidiyasa, K.; Arifin, Z. 1586 : 4; Siew Wei Hoe 125 : 4; Simpson, D.A. 2306 : 4; Sinclair, J. SFN 38688 : 4; Sino-Japanese expedition 9391 : 8; T 273 : 8; Sino-Soviet joint mission in Yunnan 2439 : 8; 3798 : 8; 4494 : 8; Sledge, W.A. 1106 : 3; 1790 : 5; Sleumer, H.O.; Vink, W. BW 14084 : 4; BW 14245 : 4; Smith, A.C. 283 : 4; 1870 : 4; 1975 : 4; 4695 : 4; 4811 : 4; 5162 : 4; 5163 : 5; 5423 : 4; 6104 : 4; 8369 : 4; 8502 : 4; 8678 : 4; Smith, E. 894 : 4; 1073 : 3; 1074 : 3; 1281 : 6; 1283 : 6; Smith, L.S. 4602 : 3; Soegeng, W. 433 : 4; Soejarto, J.J.; Fernando, O.; Sagcal, E. 8874 : 4; Soepadmo, E.; Mahmud 1091 : 4; Steenis, C.G.G.J. van 3688 : 4; 18293 : 3; 18504 : 2; 18505 : 4; 20870 : 8; Stevens, P.F. LAE 50373 : 4; LAE 58317 : 9; LAE 58759 : 9; Stevens, P.F.; Lelean, Y. LAE 58693 : 4; Stewart, R.R. 15949 : 8; Stone, B.C.; Price, M.G. 12190 : 4; Stone, B.C.; Reynoso; Sagcal, E. PPI 447 : 4; Streimann, H. N.G.F. 35860 : 5; NGF 28939 : 4; Streimann, H.; Kairo, A. NGF 39009 : 4; NGF 44042 : 9; Stresemann, E. 9 : 4; 12 : 9; 365 : 9; 405 : 9; 405 a: 5; Strugnell, E.J. 14611 : 4; Sulit, M.D. 1211 : 4; 5376 : 4; Sun Hong Fan Herb 9434 : 3; Surbeck, H. 152 : 4; 600 : 3; 1036 : 4; 1093 : 4; Survey team who collected herbs in Tibet 1102 : 8; 1102 : 8; 1139 : 8; Swart, J.W. 2448 : 4.

Tagawa, M. 542 : 8; T 634 : 3; T 646 : 2; T 1524 : 3; T 9311 : 3; Takeuchi, W. 5340 : 9; 5390 : 9; 6229 : 4; 6317 : 9; 6318 : 4; 8920 : 4; 9234 : 4; 10703 : 5; 10758 : 4; 10775 : 4; 10814 : 9; 11714 : 4; 12970 : 4; Takeuchi, W.; Towati, A.; Ama, D. 19917 : 5; Teck, L.S. S 68652 : 4; Thwaites, G.H.K. CP 1378 : 3; Ting, K.C. 796 : 2; Topping, D.L. 450 : 4; 850 : 4; 1338 : 4; 1856 : 4; Tsiang, Y. 4731 : 2; Tsoong, K.K. 1284 : 2; Turnau, E.A. 846 : 4.

Ueda, K.; Darnaedi, D. B 8941 : 4; University of San Carlos 79 : 4; 120 : 4; Unknown 46-2 : 4; 56 : 8; 60 : 2; 4699 : 8; 13924 : 4; 15513 : 4; Utteridge, T.M.A. 381 : 5.

Vaupel, F. 337 : 4; Vegetation team Qinghai-Tibet group 2503 : 8; Veldkamp, J.F. 8446 : 4; Versteeg, G.M. 1335 : 4; Versteegh, C. BW 10299 : 4; BW 10419 : 4; BW 10420 : 5; BW 10424 : 9; BW 12561 : 4; Vink, W. 17596 : 5; Vogel, E.F. de 1939 : 4; Vogel, E.F. de; Vermeulen, J.J. 7310 : 4; 7561 : 4; Voogd, C.N.A. de 128 : 4; 2498 : 3; Vriese, W.H. de 6 : 3; 25 : 3; 26 : 4; 28 : 3; 35 : 4; 66 : 4; 342 : 3.

Wagner, W.L. 6225 : 5; Waitz, F.A.C. 27 : 4; Walker, T.G. 8042 : 9; 8265 : 9; T 7541 : 5; Wallich, N. 373 : 8; Wang, C.W. 39287 : 3; 39445 : 8; 74763 : 6; 78515 : 3; 87504 : 2; Wang, W.T. 10419 : 6; Wang, Z.R. C 621 : 2; Watthana, S. QBG 21791 : 3; Webster, G.L.; Hildreth, R. 14181 : 4; Werner, E. ROS 28 : 9; Western China Academy of Sciences 4699 : 8; Whistler, A. 3549 : 4; Whitford, H.N. 999 : 4; 2248 : 4; Whitmore, T.C. BSIP 997 : 9; BSIP 2133 : 9; Whitmore, T.C.; Womersley, J.S. BSIP 688 : 9; Widjaja, E.A. 4207 : 4; Wilde, A.G. de; Vervoort, W. 530 : 4; Wilde, W.J.J.O. de; Wilde-Duyfjes, B.E.E. de 12942 : 4; 13787 : 4; 15022 : 4; Williams, K. 1981 : 4; Wilson, E.H. 5246 : 8; Winckel, W.F. 967 B: 4; 1359 : 4; 1656 B: 4; Winkler, H. 467 : 4; 1078 : 4; Wiriadinata, H. 144 : 4; Womersley, J.S.; Millar, A. NGF 7775 : 4; NGF 8377 : 4; Wong, K.M. 459 : 4; 1122 : 4; 1444 : 4; FRI 32229 : 4;; Wood, K.R. 4442 : 5; 4583 : 5; 6387 : 5; Worthington, R.D. 12919 : 4; Wray, L. 585 : 4; 1601 : 4; 3729 : 4; 5357 : 4; Wuzhishan Fern Survey 217 : 3; Wyatt-Smith, J. KEP 78837 : 4.

Xinggong Xia Xia 5419 : 8; 5520 : 8; Xu 4 : 8.

Y. Tsiang 11574 : 8; Yahud; Mahmud; S 88396 : 1; Yates, H.S. 2899 : 4; Yii Puan Ching SAR 48495 : 4; Yoshida, S. 1552 : 4; Yan Yue-Hong 3101 : 2; 12943 : 2; Yu, T.T. 16648 : 8; 17303 : 8; 17305 : 8.

Zhang Xian-Chun 106 : 2; 2355 : 3; Zhang Xian-Chun; Chen Zhen Chuan 3781 : 2; Zhang Xian-Chun; Dong Shiyong 1404 : 6; 1423 : 6; Zhongshan University 23749 : 8; Zhu Taiping 189 : 6; 476 : 6; Zhu Weiming 2705 : 8; Zollinger, H. 507 : 3; 1306 : 4; 1306 ?: 4; 1306 a: 3; 1306 B: 4; 1993 : 3.
